# Correction: Sixteen-year trends in multiple lifestyle risk behaviours by socioeconomic status from 2004 to 2019 in New South Wales, Australia

**DOI:** 10.1371/journal.pgph.0002138

**Published:** 2023-06-29

**Authors:** The PLOS Global Public Health Staff

## Notice of republication

The fifth author’s name was spelled incorrectly. This article was republished on June 01, 2023 to correct for this error. Please download this article again to view the correct version.

## Supporting information

S1 File Originally published, uncorrected article. (PDF)

S2 File Republished, corrected article. (PDF)
