# Supplementary material for: Sixteen-year trends in multiple lifestyle risk behaviours by socioeconomic status from 2004 to 2019 in New South Wales, Australia
Source: PLOS Glob Public Health. 2023 Feb 15;3(2):e0001606. doi: 10.1371/journal.pgph.0001606 (PMC10021655; doi:10.1371/journal.pgph.0001606)
Supplement: S1 File — (DOCX) [file pgph.0001606.s001.docx]

| Risk factor | Question Code | Question(s) from 2019 NSW Population Health Survey Questionnaire |
| --- | --- | --- |
| Alcohol | ALC1 | How often do you usually drink alcohol? (days per week / days per month / less than once per month / don’t drink alcohol) |
|  | ALC2 | Alcoholic drinks are measured in terms of a "standard drink". A standard drink is equal to 1 middy of full-strength beer, 1 schooner of light beer, 1 small glass of wine or 1 pub-sized nip of spirits. On a day when you drink alcohol, how many standard drinks do you usually have? (number of drinks per day) |
| Smoking | SMK1 | Which of the following best describes your smoking status? (Smoke daily / Smoke occasionally / Don't smoke now, but used to / Tried it a few times but never smoked regularly / Never smoked) |
|  | SMKEL1 | Which of the following best describes how often you use electronic cigarettes? (Never used electronic cigarettes / Tried electronic cigarettes a few times but never used them regularly / Don't use electronic cigarettes now, but used to / Use electronic cigarettes occasionally / Use electronic cigarettes daily) |
| Dietary intake | NUT1 | How many serves of vegetables do you usually eat each day? One serve is half a cup of cooked or 1 cup of salad vegetables. (serves per day) |
|  | NUT2 | How many serves of fruit do you usually eat each day? A serve is 1 medium piece or 2 small pieces of fruit or 1 cup of diced pieces. (serves per day) |
| Sugar-sweetened beverage consumption | CNFI15x | How many cups of sugar sweetened drinks such as soft drinks, cordials, sports drinks, energy drinks or iced teas do you usually drink in a day? 1 cup=250ml. One can of soft drink = 1.5 cups. One 500ml bottle of Gatorade = 2 cups. (cups per day / cups per week / cups per month / doesn’t drink sugar sweetened drinks)* |

**S1 File.** **Questions on lifestyle behaviours from the 2019 NSW Population Health Survey Questionnaire.**

*Prior to 2018, this question was worded as: “How many cups of soft drink, cordials or sports drink, such as lemonade or Gatorade, do you

usually drink in a day?”
