# Supplementary material for: Sixteen-year trends in multiple lifestyle risk behaviours by socioeconomic status from 2004 to 2019 in New South Wales, Australia
Source: PLOS Glob Public Health. 2023 Feb 15;3(2):e0001606. doi: 10.1371/journal.pgph.0001606 (PMC10021655; doi:10.1371/journal.pgph.0001606)
Supplement: S2 File — (DOCX) [file pgph.0001606.s002.docx]

**S2 File.** **Missing data.**

The data contained a relatively large number of missing data points, both because not all participants answered every question in each survey, but also because not every question was *asked* in each year of the study, with most missingness due to the latter. As such, while the percentages of missing information in the data were relatively low (9% of data points were missing), only 33% of cases contained complete data, largely due to missingness in use of electronic cigarettes and sugar-sweetened beverages, which were not asked in all waves. Frequencies and the ten most common patterns of missing data are reported in Table S1.

Data was confirmed to be not missing completely at random via Little’s test (1). Because the majority of missing data was caused by questions not being asked in specific waves, we assumed missingness was not missing not at random. As such, we have assumed the data to be missing at random. Because missingness can introduce bias when there is missingness in the outcome variables (2) in models, the analyses were conducted using multiple imputation. We conducted the imputation using fully conditional specification (also called chained equations) (3) in R (4), using the R package ‘mice’ (5). We specified the imputation using predictive mean matching (6) for continuous variables, and random forests (7) for all other variables. We used M=40 imputations (8). Analyses were then conducted on each imputed dataset, and combined using Rubin’s rules (9).

### References

1. Little RJ. A test of missing completely at random for multivariate data with missing values. Journal of the American statistical Association. 1988;83(404):1198-202.

2. Hughes RA, Heron J, Sterne JAC, Tilling K. Accounting for missing data in statistical analyses: multiple imputation is not always the answer. International Journal of Epidemiology. 2019;48(4):1294-304.

3. White IR, Royston P, Wood AM. Multiple imputation using chained equations: issues and guidance for practice. Statistics in medicine. 2011;30(4):377-99.

4. R Core Team. R: A Language and Environment for Statistical Computing. Vienna, Austria: R Foundation for Statistical Computing; 2022.

5. Van Buuren S, Groothuis-Oudshoorn K. mice: Multivariate imputation by chained equations in R. Journal of statistical software. 2011;45:1-67.

6. Landerman LR, Land KC, Pieper CF. An empirical evaluation of the predictive mean matching method for imputing missing values. Sociological methods & research. 1997;26(1):3-33.

7. Shah AD, Bartlett JW, Carpenter J, Nicholas O, Hemingway H. Comparison of random forest and parametric imputation models for imputing missing data using MICE: a CALIBER study. American journal of epidemiology. 2014;179(6):764-74.

8. Graham JW, Olchowski AE, Gilreath TD. How many imputations are really needed? Some practical clarifications of multiple imputation theory. Prevention Science. 2007;8(3):206-13.

9. Rubin DB. Multiple imputation for nonresponse in surveys. John Wiley & Sons, 2004, Vol. 81.
