# Supplementary material for: Sixteen-year trends in multiple lifestyle risk behaviours by socioeconomic status from 2004 to 2019 in New South Wales, Australia
Source: PLOS Glob Public Health. 2023 Feb 15;3(2):e0001606. doi: 10.1371/journal.pgph.0001606 (PMC10021655; doi:10.1371/journal.pgph.0001606)
Supplement: S1 Fig — (DOCX) [file pgph.0001606.s003.docx]

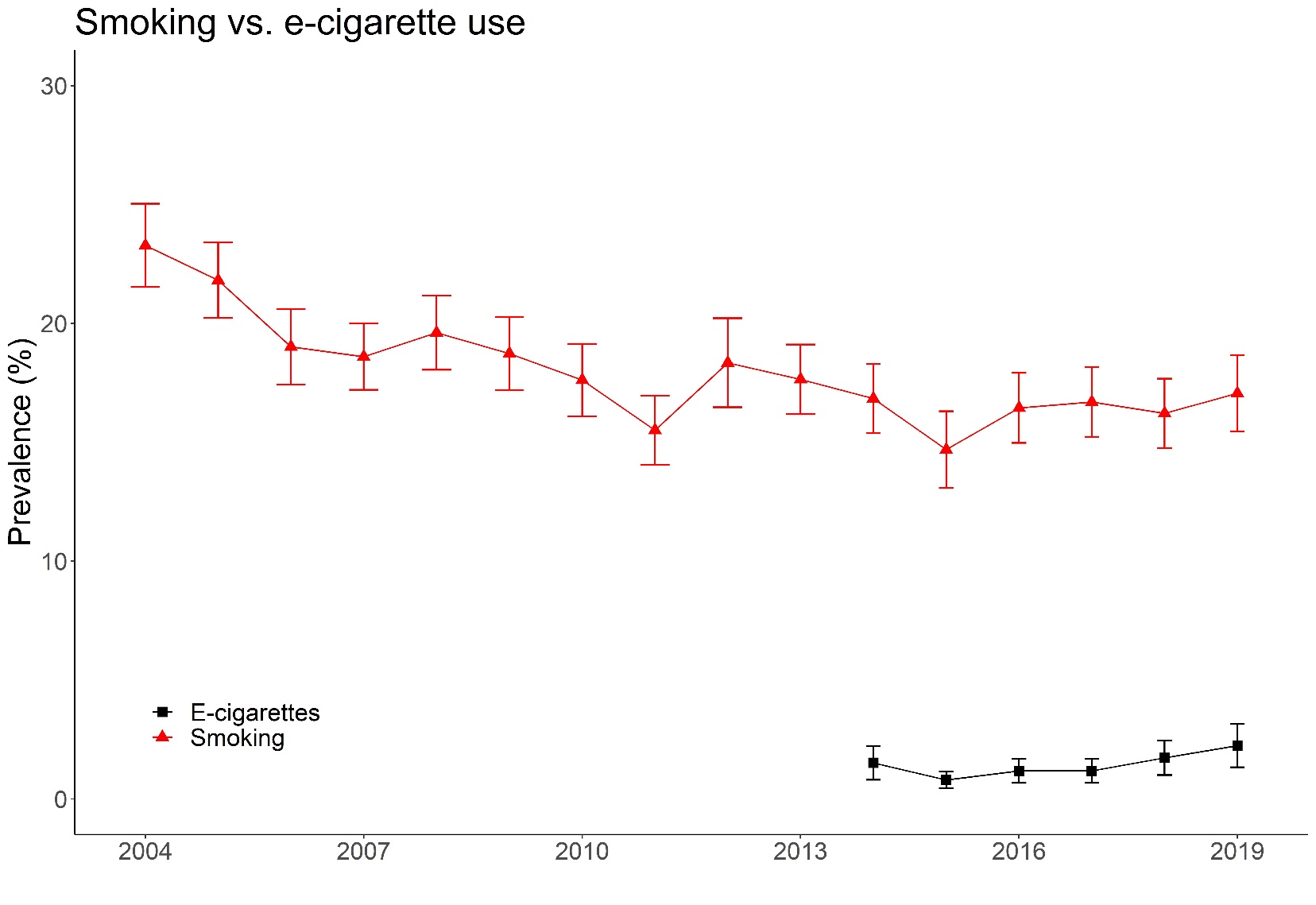


**S1 Fig. Prevalence of being a current tobacco smoker and prevalence of current e-cigarette use* by year in NSW adults aged 16 years and over, 2004-2019.**

*****The survey question related to e-cigarette use was only asked from 2014 onwards.
