# Supplementary material for: Sixteen-year trends in multiple lifestyle risk behaviours by socioeconomic status from 2004 to 2019 in New South Wales, Australia
Source: PLOS Glob Public Health. 2023 Feb 15;3(2):e0001606. doi: 10.1371/journal.pgph.0001606 (PMC10021655; doi:10.1371/journal.pgph.0001606)
Supplement: S2 Fig — (DOCX) [file pgph.0001606.s004.docx]

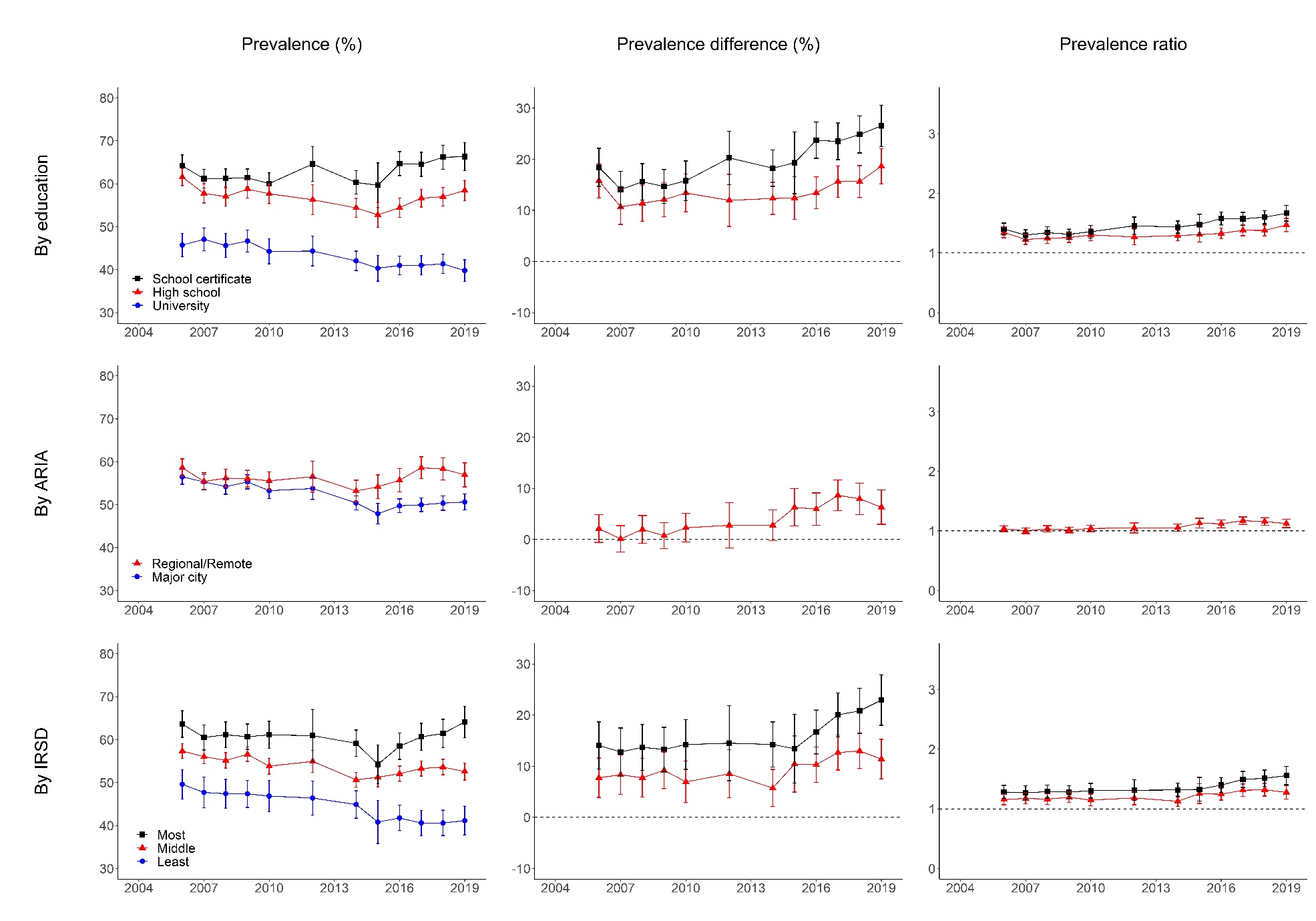


**S2 Fig. Prevalence, prevalence difference and prevalence ratio of the combined lifestyle risk index* for the most and least disadvantaged groups for the three socioeconomic indicators, by year, NSW adults aged 16 years and over, 2006-2019.**

* Based on current smoking, excessive alcohol consumption, insufficient physical activity, insufficient fruit and/or vegetable consumption, and daily sugar-sweetened beverage consumption.
