# Supplementary material for: Sixteen-year trends in multiple lifestyle risk behaviours by socioeconomic status from 2004 to 2019 in New South Wales, Australia
Source: PLOS Glob Public Health. 2023 Feb 15;3(2):e0001606. doi: 10.1371/journal.pgph.0001606 (PMC10021655; doi:10.1371/journal.pgph.0001606)
Supplement: S1 Table — (DOCX) [file pgph.0001606.s005.docx]

**S1 Table.** **Patterns of missing data.**

| **Variable name** | **Variable description** | **Missingness pattern** | | | | | | | | | | **Number missing in variable** |
| --- | --- | --- | --- | --- | --- | --- | --- | --- | --- | --- | --- | --- |
|  |  | **(1)** | **(2)** | **(3)** | **(4)** | **(5)** | **(6)** | **(7)** | **(8)** | **(9)** | **(10)** |  |
| smkel1 | Used electronic cigarette | X | X | X |  |  | X | X |  | X | X | 113,206 |
| ssd_freq | Consumed sugar-sweetened drinks | X |  |  |  |  |  | X |  |  | X | 61,621 |
| walk_mins | Number of minutes of walking |  |  | X | X |  |  |  |  | X |  | 30,273 |
| bmi | Body mass index |  |  |  |  | X | X | X |  |  |  | 15,664 |
| veg_per_day | Serves of vegetables per day |  |  |  |  |  |  |  |  |  | X | 11,727 |
| vigorous_mins | Number of minutes of vigorous physical activity |  |  |  |  |  |  |  |  | X |  | 11,055 |
| moderate_mins | Number of minutes of moderate physical activity |  |  |  |  |  |  |  |  | X |  | 10,992 |
| fruit_per_day | Serves of fruit per day |  |  |  |  |  |  |  |  |  | X | 9,637 |
| new_alc_quant | Frequency of alcohol consumption |  |  |  |  |  |  |  |  |  |  | 9,487 |
| new_alc_freq | Typical quantity of alcohol consumed when drinking |  |  |  |  |  |  |  |  |  |  | 8,174 |
| smk1 | Smoking status |  |  |  |  |  |  |  |  |  |  | 7,467 |
| hdi_category | Human development index of birth country |  |  |  |  |  |  |  | X |  |  | 4,937 |
| lanpa | Language spoken at home |  |  |  |  |  |  |  |  |  |  | 4,584 |
| qallp | Highest qualification completed |  |  |  |  |  |  |  |  |  |  | 2,639 |
| lfsp | Current employment status |  |  |  |  |  |  |  |  |  |  | 1,014 |
| ariaplusc | ARIA+ remoteness category |  |  |  |  |  |  |  |  |  |  | 380 |
| seifa_irsd_2016 | SEIFA IRSD quintile |  |  |  |  |  |  |  |  |  |  | 353 |
| **Number missing in pattern** | | 44,790 | 24,600 | 10,087 | 3,980 | 2,255 | 2,149 | 1,861 | 1,780 | 1,698 | 1,316 |  |

Note: Xs shown in the table represent that data was missing for that specific variable in that ‘pattern’ of missing data. The total number of cases with each ‘pattern’ of missing data are shown in the bottom row. The number of cases with missing data for each specific variable are shown in the right-most column.
