# Supplementary material for: Sixteen-year trends in multiple lifestyle risk behaviours by socioeconomic status from 2004 to 2019 in New South Wales, Australia
Source: PLOS Glob Public Health. 2023 Feb 15;3(2):e0001606. doi: 10.1371/journal.pgph.0001606 (PMC10021655; doi:10.1371/journal.pgph.0001606)
Supplement: S2 Table — (DOCX) [file pgph.0001606.s006.docx]

**S2 Table. Prevalence (with 95% confidence intervals) of individual lifestyle risk factors and combined lifestyle risk index, by year, persons 16 years and over, 2004-2019, NSW, Australia.**

| **Lifestyle risk factor** | **Year** | **Estimate** | **Standard error** | **95% CI, lower** | **95% CI, upper** |
| --- | --- | --- | --- | --- | --- |
| Current smoking | 2004 | 21.8% | 0.6% | 20.7% | 22.9% |
|  | 2005 | 20.4% | 0.5% | 19.5% | 21.4% |
|  | 2006 | 17.8% | 0.6% | 16.7% | 18.9% |
|  | 2007 | 17.5% | 0.5% | 16.4% | 18.5% |
|  | 2008 | 18.4% | 0.6% | 17.3% | 19.5% |
|  | 2009 | 17.6% | 0.5% | 16.6% | 18.6% |
|  | 2010 | 16.5% | 0.5% | 15.5% | 17.6% |
|  | 2011 | 14.6% | 0.5% | 13.5% | 15.6% |
|  | 2012 | 17.2% | 0.7% | 15.8% | 18.7% |
|  | 2013 | 16.6% | 0.5% | 15.6% | 17.6% |
|  | 2014 | 16.4% | 0.5% | 15.3% | 17.4% |
|  | 2015 | 14.1% | 0.7% | 12.8% | 15.4% |
|  | 2016 | 15.9% | 0.5% | 14.8% | 16.9% |
|  | 2017 | 16.2% | 0.5% | 15.1% | 17.2% |
|  | 2018 | 15.9% | 0.5% | 14.8% | 16.9% |
|  | 2019 | 17.1% | 0.6% | 15.9% | 18.4% |
|  |  |  |  |  |  |
| Excessive alcohol consumption | 2004 | 15.4% | 0.5% | 14.5% | 16.4% |
|  | 2005 | 14.2% | 0.4% | 13.4% | 15.0% |
|  | 2006 | 15.3% | 0.5% | 14.4% | 16.3% |
|  | 2007 | 14.0% | 0.5% | 13.0% | 14.9% |
|  | 2008 | 15.6% | 0.5% | 14.6% | 16.5% |
|  | 2009 | 16.0% | 0.4% | 15.2% | 16.9% |
|  | 2010 | 13.9% | 0.5% | 13.0% | 14.8% |
|  | 2011 | 14.2% | 0.5% | 13.2% | 15.2% |
|  | 2012 | 13.4% | 0.6% | 12.3% | 14.6% |
|  | 2013 | 12.4% | 0.4% | 11.6% | 13.3% |
|  | 2014 | 13.2% | 0.5% | 12.3% | 14.1% |
|  | 2015 | 12.4% | 0.6% | 11.3% | 13.6% |
|  | 2016 | 13.7% | 0.5% | 12.8% | 14.7% |
|  | 2017 | 14.0% | 0.5% | 13.1% | 14.9% |
|  | 2018 | 14.5% | 0.5% | 13.6% | 15.5% |
|  | 2019 | 13.7% | 0.5% | 12.7% | 14.7% |
|  |  |  |  |  |  |
| Insufficient physical activity | 2004 | 39.1% | 0.7% | 37.8% | 40.4% |
|  | 2005 | 38.5% | 0.6% | 37.4% | 39.7% |
|  | 2006 | 37.0% | 0.7% | 35.7% | 38.3% |
|  | 2007 | 36.6% | 0.7% | 35.2% | 38.0% |
|  | 2008 | 36.2% | 0.7% | 34.9% | 37.6% |
|  | 2009 | 35.1% | 0.6% | 34.0% | 36.3% |
|  | 2010 | 36.0% | 0.7% | 34.6% | 37.3% |
|  | 2011 | 36.9% | 0.7% | 35.6% | 38.3% |
|  | 2012 | 39.9% | 1.1% | 37.8% | 42.1% |
|  | 2013 | 37.2% | 0.6% | 36.0% | 38.4% |
|  | 2014 | 33.4% | 0.7% | 32.1% | 34.7% |
|  | 2015 | 32.9% | 0.9% | 31.2% | 34.6% |
|  | 2016 | 33.4% | 0.6% | 32.1% | 34.6% |
|  | 2017 | 33.5% | 0.6% | 32.3% | 34.8% |
|  | 2018 | 31.8% | 0.6% | 30.6% | 33.1% |
|  | 2019 | 30.9% | 0.7% | 29.5% | 32.3% |
|  |  |  |  |  |  |
| Insufficient fruit and/or vegetable consumption | 2004 | 79.0% | 0.5% | 78.0% | 80.1% |
|  | 2005 | 75.8% | 0.5% | 74.9% | 76.8% |
|  | 2006 | 73.6% | 0.6% | 72.5% | 74.8% |
|  | 2007 | 73.0% | 0.6% | 71.9% | 74.2% |
|  | 2008 | 72.6% | 0.6% | 71.4% | 73.8% |
|  | 2009 | 70.9% | 0.6% | 69.8% | 72.0% |
|  | 2010 | 72.5% | 0.6% | 71.4% | 73.7% |
|  | 2011 | 73.5% | 0.6% | 72.3% | 74.7% |
|  | 2012 | 73.7% | 0.9% | 72.1% | 75.4% |
|  | 2013 | 74.0% | 0.6% | 72.9% | 75.1% |
|  | 2014 | 74.7% | 0.6% | 73.6% | 75.9% |
|  | 2015 | 78.4% | 0.7% | 77.0% | 79.8% |
|  | 2016 | 77.0% | 0.6% | 75.8% | 78.2% |
|  | 2017 | 78.8% | 0.6% | 77.7% | 79.9% |
|  | 2018 | 81.2% | 0.5% | 80.1% | 82.3% |
|  | 2019 | 81.7% | 0.6% | 80.6% | 82.9% |
|  |  |  |  |  |  |
| Daily sugar-sweetened beverage consumption | 2004 | - | - | - | - |
|  | 2005 | - | - | - | - |
|  | 2006 | 29.9% | 0.7% | 28.6% | 31.2% |
|  | 2007 | 27.5% | 0.6% | 26.3% | 28.6% |
|  | 2008 | 27.6% | 0.7% | 26.3% | 28.9% |
|  | 2009 | 29.7% | 0.7% | 28.4% | 31.0% |
|  | 2010 | 27.0% | 0.8% | 25.5% | 28.5% |
|  | 2011 | - | - | - | - |
|  | 2012 | 23.3% | 0.7% | 21.9% | 24.7% |
|  | 2013 | - | - | - | - |
|  | 2014 | 22.9% | 0.6% | 21.7% | 24.1% |
|  | 2015 | 20.2% | 0.7% | 18.8% | 21.5% |
|  | 2016 | 21.0% | 0.6% | 19.8% | 22.2% |
|  | 2017 | 21.2% | 0.6% | 20.0% | 22.3% |
|  | 2018 | 21.3% | 0.6% | 20.2% | 22.5% |
|  | 2019 | 21.2% | 0.7% | 19.9% | 22.5% |
|  |  |  |  |  |  |
| High total lifestyle risk^a^ | 2004 | 50.4% | 0.7% | 49.0% | 51.8% |
|  | 2005 | 47.6% | 0.6% | 46.4% | 48.8% |
|  | 2006 | 45.1% | 0.7% | 43.7% | 46.5% |
|  | 2007 | 43.7% | 0.7% | 42.3% | 45.2% |
|  | 2008 | 44.2% | 0.7% | 42.8% | 45.6% |
|  | 2009 | 43.4% | 0.7% | 42.1% | 44.7% |
|  | 2010 | 42.9% | 0.7% | 41.4% | 44.3% |
|  | 2011 | 43.5% | 0.7% | 42.0% | 44.9% |
|  | 2012 | 45.6% | 1.1% | 43.5% | 47.8% |
|  | 2013 | 43.8% | 0.7% | 42.5% | 45.1% |
|  | 2014 | 42.2% | 0.7% | 40.8% | 43.6% |
|  | 2015 | 41.8% | 1.0% | 40.0% | 43.7% |
|  | 2016 | 42.5% | 0.7% | 41.1% | 43.9% |
|  | 2017 | 43.5% | 0.7% | 42.2% | 44.9% |
|  | 2018 | 43.9% | 0.7% | 42.5% | 45.3% |
|  | 2019 | 43.7% | 0.8% | 42.2% | 45.3% |
|  |  |  |  |  |  |
| High total lifestyle risk (supplementary analysis)^b^ | 2004 | - | - | - | - |
|  | 2005 | - | - | - | - |
|  | 2006 | 57.0% | 0.7% | 55.6% | 58.4% |
|  | 2007 | 55.3% | 0.7% | 53.9% | 56.7% |
|  | 2008 | 54.7% | 0.7% | 53.3% | 56.1% |
|  | 2009 | 55.5% | 0.7% | 54.1% | 56.8% |
|  | 2010 | 53.9% | 0.8% | 52.4% | 55.3% |
|  | 2011 | - | - | - | - |
|  | 2012 | 54.5% | 1.1% | 52.4% | 56.6% |
|  | 2013 | - | - | - | - |
|  | 2014 | 51.2% | 0.7% | 49.8% | 52.5% |
|  | 2015 | 49.5% | 1.0% | 47.5% | 51.4% |
|  | 2016 | 51.2% | 0.7% | 49.8% | 52.6% |
|  | 2017 | 52.0% | 0.7% | 50.6% | 53.3% |
|  | 2018 | 52.3% | 0.7% | 50.9% | 53.7% |
|  | 2019 | 52.2% | 0.8% | 50.6% | 53.7% |

Abbreviations: CI, confidence interval.

^a^ Defined as engaging in two or more lifestyle risk behaviours, based on the following four individual risk behaviours: excessive alcohol consumption, insufficient physical activity, insufficient fruit and/or vegetable consumption, and current smoking.

^b^ Defined as engaging in two or more lifestyle risk behaviours, based on the following five individual risk behaviours: excessive alcohol consumption, insufficient physical activity, insufficient fruit and/or vegetable consumption, current smoking, and daily sugar-sweetened beverage consumption.
