# Supplementary material for: Sixteen-year trends in multiple lifestyle risk behaviours by socioeconomic status from 2004 to 2019 in New South Wales, Australia
Source: PLOS Glob Public Health. 2023 Feb 15;3(2):e0001606. doi: 10.1371/journal.pgph.0001606 (PMC10021655; doi:10.1371/journal.pgph.0001606)
Supplement: S3 Table — (DOCX) [file pgph.0001606.s007.docx]

**S3 Table. Prevalence, prevalence differences and prevalence ratios of individual lifestyle risk factors and combined lifestyle risk index by educational attainment, by year, persons 16 years and over, 2004-2019, NSW, Australia.**

| **Lifestyle risk factor** | **Year** | **University** | **School certificate or less** | | | **Higher school certificate or trade** | | |
| --- | --- | --- | --- | --- | --- | --- | --- | --- |
|  |  | **Prevalence %  (95% CI)** | **Prevalence %  (95% CI)** | **Risk difference*  (95% CI)** | **Relative risk*  (95% CI)** | **Prevalence %  (95% CI)** | **Risk difference*  (95% CI)** | **Relative risk*  (95% CI)** |
| Current smoking | 2004 | 15.70 (13.66, 17.74) | 26.94 (24.57, 29.31) | 11.24 (8.11, 14.38) | 1.72 (1.45, 1.99) | 23.45 (21.76, 25.13) | 7.75 (5.10, 10.40) | 1.49 (1.27, 1.72) |
|  | 2005 | 13.61 (12.00, 15.21) | 25.08 (23.00, 27.15) | 11.47 (8.84, 14.10) | 1.84 (1.58, 2.11) | 22.88 (21.38, 24.38) | 9.27 (7.07, 11.48) | 1.68 (1.45, 1.91) |
|  | 2006 | 11.28 (9.58, 12.97) | 24.01 (21.43, 26.59) | 12.73 (9.64, 15.83) | 2.13 (1.73, 2.52) | 19.97 (18.30, 21.64) | 8.69 (6.31, 11.08) | 1.77 (1.47, 2.08) |
|  | 2007 | 11.51 (9.80, 13.23) | 22.55 (20.58, 24.53) | 11.04 (8.42, 13.65) | 1.96 (1.62, 2.30) | 18.88 (17.18, 20.58) | 7.36 (4.96, 9.77) | 1.64 (1.36, 1.93) |
|  | 2008 | 12.04 (10.30, 13.77) | 23.92 (21.81, 26.03) | 11.88 (9.12, 14.64) | 1.99 (1.65, 2.33) | 19.68 (17.89, 21.46) | 7.64 (5.16, 10.11) | 1.64 (1.36, 1.91) |
|  | 2009 | 11.91 (10.24, 13.58) | 24.19 (22.20, 26.18) | 12.28 (9.68, 14.89) | 2.03 (1.70, 2.36) | 18.30 (16.63, 19.97) | 6.39 (4.03, 8.76) | 1.54 (1.28, 1.80) |
|  | 2010 | 9.78 (8.15, 11.41) | 24.18 (21.91, 26.46) | 14.41 (11.60, 17.21) | 2.47 (2.00, 2.95) | 17.49 (15.82, 19.16) | 7.72 (5.38, 10.05) | 1.79 (1.44, 2.13) |
|  | 2011 | 9.31 (7.72, 10.91) | 22.46 (20.12, 24.81) | 13.15 (10.32, 15.99) | 2.41 (1.93, 2.90) | 14.73 (13.04, 16.42) | 5.42 (3.09, 7.74) | 1.58 (1.26, 1.91) |
|  | 2012 | 11.03 (8.64, 13.41) | 24.33 (20.90, 27.77) | 13.31 (9.13, 17.48) | 2.21 (1.64, 2.78) | 18.07 (15.89, 20.26) | 7.05 (3.81, 10.29) | 1.64 (1.23, 2.05) |
|  | 2013 | 10.58 (9.10, 12.06) | 22.65 (20.18, 25.13) | 12.07 (9.19, 14.96) | 2.14 (1.76, 2.52) | 18.48 (16.90, 20.06) | 7.89 (5.72, 10.07) | 1.75 (1.46, 2.03) |
|  | 2014 | 9.93 (8.58, 11.29) | 22.06 (19.49, 24.63) | 12.12 (9.21, 15.04) | 2.22 (1.82, 2.62) | 19.24 (17.49, 20.98) | 9.30 (7.08, 11.52) | 1.94 (1.62, 2.25) |
|  | 2015 | 9.11 (7.42, 10.81) | 23.42 (19.29, 27.55) | 14.30 (9.83, 18.77) | 2.57 (1.91, 3.23) | 14.49 (12.72, 16.26) | 5.37 (2.92, 7.82) | 1.59 (1.24, 1.94) |
|  | 2016 | 9.71 (8.40, 11.02) | 26.34 (23.42, 29.26) | 16.63 (13.42, 19.84) | 2.71 (2.24, 3.19) | 17.18 (15.39, 18.98) | 7.47 (5.25, 9.69) | 1.77 (1.47, 2.07) |
|  | 2017 | 8.80 (7.50, 10.11) | 24.21 (21.21, 27.21) | 15.41 (12.12, 18.69) | 2.75 (2.22, 3.28) | 19.82 (18.06, 21.59) | 11.02 (8.82, 13.23) | 2.25 (1.86, 2.64) |
|  | 2018 | 10.79 (9.36, 12.23) | 23.86 (20.84, 26.88) | 13.06 (9.71, 16.41) | 2.21 (1.80, 2.62) | 17.67 (15.94, 19.39) | 6.87 (4.62, 9.12) | 1.64 (1.37, 1.91) |
|  | 2019 | 10.69 (9.07, 12.32) | 25.93 (22.39, 29.47) | 15.23 (11.33, 19.13) | 2.42 (1.93, 2.92) | 20.04 (18.04, 22.04) | 9.35 (6.77, 11.92) | 1.87 (1.53, 2.21) |
| Excessive alcohol consumption | 2004 | 13.69 (11.87, 15.51) | 14.95 (13.23, 16.66) | 1.26 (-1.25, 3.76) | 1.09 (0.90, 1.28) | 16.68 (15.24, 18.11) | 2.99 (0.67, 5.31) | 1.22 (1.03, 1.41) |
|  | 2005 | 15.02 (13.42, 16.61) | 13.81 (12.19, 15.42) | -1.21 (-3.49, 1.06) | 0.92 (0.77, 1.06) | 13.87 (12.76, 14.98) | -1.15 (-3.10, 0.80) | 0.92 (0.80, 1.05) |
|  | 2006 | 15.65 (13.74, 17.56) | 14.57 (12.69, 16.44) | -1.08 (-3.76, 1.60) | 0.93 (0.77, 1.10) | 15.52 (14.11, 16.93) | -0.13 (-2.51, 2.25) | 0.99 (0.84, 1.14) |
|  | 2007 | 14.80 (12.97, 16.63) | 12.30 (10.79, 13.82) | -2.50 (-4.86, -0.14) | 0.83 (0.69, 0.98) | 14.57 (13.09, 16.04) | -0.23 (-2.55, 2.08) | 0.99 (0.83, 1.14) |
|  | 2008 | 16.74 (14.82, 18.66) | 14.91 (13.32, 16.49) | -1.83 (-4.31, 0.65) | 0.89 (0.75, 1.03) | 15.26 (13.73, 16.79) | -1.48 (-3.92, 0.96) | 0.91 (0.77, 1.05) |
|  | 2009 | 15.61 (13.99, 17.23) | 14.74 (13.32, 16.16) | -0.87 (-3.04, 1.30) | 0.94 (0.81, 1.08) | 17.23 (15.75, 18.70) | 1.62 (-0.58, 3.82) | 1.10 (0.95, 1.25) |
|  | 2010 | 13.55 (11.76, 15.35) | 12.78 (11.27, 14.30) | -0.77 (-3.12, 1.58) | 0.94 (0.78, 1.11) | 14.75 (13.32, 16.17) | 1.19 (-1.10, 3.49) | 1.09 (0.91, 1.27) |
|  | 2011 | 14.37 (12.44, 16.30) | 12.85 (11.38, 14.33) | -1.52 (-3.96, 0.91) | 0.89 (0.74, 1.05) | 14.89 (13.34, 16.45) | 0.52 (-1.96, 3.00) | 1.04 (0.86, 1.21) |
|  | 2012 | 12.92 (11.06, 14.79) | 13.64 (11.46, 15.81) | 0.71 (-2.15, 3.58) | 1.06 (0.83, 1.28) | 13.66 (11.73, 15.60) | 0.74 (-1.94, 3.43) | 1.06 (0.84, 1.27) |
|  | 2013 | 11.23 (9.81, 12.65) | 11.31 (9.70, 12.92) | 0.08 (-2.07, 2.23) | 1.01 (0.82, 1.20) | 13.79 (12.42, 15.16) | 2.56 (0.59, 4.54) | 1.23 (1.03, 1.43) |
|  | 2014 | 12.92 (11.41, 14.44) | 10.90 (9.29, 12.52) | -2.02 (-4.24, 0.19) | 0.84 (0.68, 1.00) | 14.71 (13.20, 16.21) | 1.78 (-0.35, 3.92) | 1.14 (0.96, 1.32) |
|  | 2015 | 11.79 (9.65, 13.93) | 10.68 (9.05, 12.32) | -1.11 (-3.81, 1.59) | 0.91 (0.69, 1.12) | 13.86 (12.08, 15.64) | 2.07 (-0.72, 4.86) | 1.18 (0.91, 1.44) |
|  | 2016 | 13.15 (11.66, 14.65) | 12.52 (10.57, 14.48) | -0.63 (-3.09, 1.83) | 0.95 (0.77, 1.14) | 14.87 (13.36, 16.37) | 1.71 (-0.42, 3.84) | 1.13 (0.96, 1.30) |
|  | 2017 | 12.39 (10.94, 13.85) | 13.40 (11.51, 15.30) | 1.01 (-1.38, 3.40) | 1.08 (0.88, 1.28) | 15.44 (14.01, 16.86) | 3.05 (1.01, 5.09) | 1.25 (1.06, 1.43) |
|  | 2018 | 14.90 (13.29, 16.52) | 12.20 (10.36, 14.04) | -2.70 (-5.15, -0.26) | 0.82 (0.67, 0.97) | 15.10 (13.64, 16.55) | 0.19 (-1.98, 2.37) | 1.01 (0.87, 1.16) |
|  | 2019 | 13.00 (11.31, 14.69) | 12.61 (10.55, 14.68) | -0.39 (-3.05, 2.28) | 0.97 (0.77, 1.17) | 14.61 (13.17, 16.05) | 1.61 (-0.61, 3.83) | 1.12 (0.94, 1.31) |
| Insufficient physical activity | 2004 | 33.68 (31.15, 36.21) | 43.43 (41.05, 45.82) | 9.75 (6.26, 13.24) | 1.29 (1.17, 1.41) | 40.34 (38.40, 42.27) | 6.66 (3.47, 9.84) | 1.20 (1.09, 1.30) |
|  | 2005 | 32.12 (30.02, 34.22) | 42.84 (40.67, 45.01) | 10.72 (7.70, 13.75) | 1.33 (1.22, 1.44) | 40.65 (38.95, 42.34) | 8.53 (5.82, 11.23) | 1.27 (1.17, 1.36) |
|  | 2006 | 30.72 (28.34, 33.10) | 43.15 (40.53, 45.78) | 12.44 (8.90, 15.98) | 1.41 (1.27, 1.54) | 38.53 (36.52, 40.55) | 7.82 (4.69, 10.94) | 1.25 (1.14, 1.37) |
|  | 2007 | 33.80 (31.22, 36.39) | 40.04 (37.79, 42.29) | 6.24 (2.90, 9.58) | 1.19 (1.07, 1.30) | 36.35 (34.20, 38.50) | 2.55 (-0.76, 5.86) | 1.08 (0.97, 1.18) |
|  | 2008 | 32.70 (30.28, 35.12) | 39.68 (37.51, 41.84) | 6.98 (3.68, 10.28) | 1.21 (1.10, 1.33) | 36.62 (34.48, 38.76) | 3.93 (0.72, 7.13) | 1.12 (1.02, 1.23) |
|  | 2009 | 31.24 (29.02, 33.45) | 39.20 (37.20, 41.19) | 7.96 (4.97, 10.95) | 1.25 (1.14, 1.36) | 35.67 (33.71, 37.62) | 4.43 (1.47, 7.39) | 1.14 (1.04, 1.24) |
|  | 2010 | 31.99 (29.50, 34.48) | 39.82 (37.58, 42.05) | 7.83 (4.48, 11.17) | 1.24 (1.13, 1.36) | 36.82 (34.74, 38.89) | 4.83 (1.59, 8.06) | 1.15 (1.04, 1.26) |
|  | 2011 | 32.88 (30.52, 35.24) | 40.72 (38.45, 43.00) | 7.85 (4.58, 11.11) | 1.24 (1.13, 1.35) | 38.02 (35.73, 40.31) | 5.14 (1.84, 8.44) | 1.16 (1.05, 1.27) |
|  | 2012 | 33.71 (30.20, 37.23) | 46.77 (41.92, 51.62) | 13.05 (7.40, 18.71) | 1.39 (1.20, 1.58) | 40.74 (37.17, 44.31) | 7.03 (1.71, 12.34) | 1.21 (1.04, 1.38) |
|  | 2013 | 32.95 (30.95, 34.95) | 43.89 (41.38, 46.40) | 10.94 (7.73, 14.15) | 1.33 (1.22, 1.44) | 37.17 (35.28, 39.06) | 4.22 (1.47, 6.97) | 1.13 (1.04, 1.22) |
|  | 2014 | 27.97 (25.95, 29.98) | 39.12 (36.44, 41.79) | 11.15 (7.79, 14.51) | 1.40 (1.26, 1.54) | 35.24 (33.15, 37.34) | 7.27 (4.36, 10.19) | 1.26 (1.14, 1.38) |
|  | 2015 | 26.93 (24.48, 29.38) | 37.89 (33.73, 42.06) | 10.96 (6.11, 15.82) | 1.41 (1.21, 1.61) | 36.07 (33.26, 38.89) | 9.14 (5.38, 12.90) | 1.34 (1.18, 1.50) |
|  | 2016 | 27.79 (25.90, 29.68) | 40.28 (37.57, 42.99) | 12.49 (9.18, 15.80) | 1.45 (1.31, 1.59) | 35.41 (33.27, 37.54) | 7.62 (4.77, 10.46) | 1.27 (1.16, 1.39) |
|  | 2017 | 27.91 (25.95, 29.88) | 41.33 (38.40, 44.26) | 13.41 (9.91, 16.92) | 1.48 (1.33, 1.63) | 35.34 (33.35, 37.33) | 7.42 (4.61, 10.24) | 1.27 (1.15, 1.38) |
|  | 2018 | 24.57 (22.69, 26.45) | 40.92 (38.06, 43.78) | 16.35 (12.92, 19.78) | 1.67 (1.49, 1.84) | 35.20 (33.13, 37.28) | 10.63 (7.82, 13.45) | 1.43 (1.29, 1.57) |
|  | 2019 | 24.18 (22.03, 26.32) | 40.54 (37.01, 44.08) | 16.37 (12.23, 20.50) | 1.68 (1.47, 1.89) | 33.56 (31.41, 35.70) | 9.38 (6.33, 12.43) | 1.39 (1.24, 1.54) |
| Insufficient fruit and/or vegetable consumption | 2004 | 75.64 (73.39, 77.89) | 81.76 (80.06, 83.47) | 6.12 (3.30, 8.95) | 1.08 (1.04, 1.12) | 79.57 (78.04, 81.11) | 3.93 (1.21, 6.66) | 1.05 (1.01, 1.09) |
|  | 2005 | 70.34 (68.36, 72.32) | 78.31 (76.69, 79.93) | 7.98 (5.41, 10.54) | 1.11 (1.07, 1.15) | 77.99 (76.69, 79.30) | 7.66 (5.28, 10.03) | 1.11 (1.07, 1.15) |
|  | 2006 | 66.48 (64.02, 68.94) | 78.54 (76.49, 80.60) | 12.06 (8.85, 15.27) | 1.18 (1.13, 1.24) | 75.93 (74.29, 77.56) | 9.44 (6.48, 12.41) | 1.14 (1.09, 1.19) |
|  | 2007 | 67.13 (64.78, 69.49) | 77.90 (76.30, 79.49) | 10.76 (7.90, 13.62) | 1.16 (1.11, 1.21) | 73.89 (72.05, 75.74) | 6.76 (3.75, 9.77) | 1.10 (1.05, 1.15) |
|  | 2008 | 66.50 (64.03, 68.96) | 76.77 (75.10, 78.43) | 10.27 (7.34, 13.20) | 1.15 (1.11, 1.20) | 73.94 (72.12, 75.77) | 7.45 (4.39, 10.50) | 1.11 (1.06, 1.16) |
|  | 2009 | 65.46 (63.21, 67.70) | 75.30 (73.66, 76.94) | 9.84 (7.06, 12.62) | 1.15 (1.10, 1.20) | 72.12 (70.32, 73.91) | 6.66 (3.78, 9.54) | 1.10 (1.05, 1.15) |
|  | 2010 | 67.35 (64.91, 69.79) | 76.42 (74.62, 78.23) | 9.07 (6.02, 12.12) | 1.13 (1.09, 1.18) | 73.98 (72.17, 75.80) | 6.63 (3.57, 9.68) | 1.10 (1.05, 1.15) |
|  | 2011 | 67.08 (64.69, 69.46) | 77.95 (76.10, 79.79) | 10.87 (7.85, 13.89) | 1.16 (1.11, 1.21) | 75.91 (74.07, 77.76) | 8.84 (5.82, 11.86) | 1.13 (1.08, 1.18) |
|  | 2012 | 66.49 (63.06, 69.93) | 80.44 (78.34, 82.54) | 13.95 (9.92, 17.97) | 1.21 (1.14, 1.28) | 74.92 (72.31, 77.53) | 8.43 (4.11, 12.76) | 1.13 (1.06, 1.20) |
|  | 2013 | 68.64 (66.60, 70.68) | 79.56 (77.56, 81.55) | 10.91 (8.06, 13.77) | 1.16 (1.11, 1.20) | 75.05 (73.39, 76.71) | 6.41 (3.77, 9.06) | 1.09 (1.05, 1.13) |
|  | 2014 | 69.36 (67.27, 71.45) | 80.00 (77.96, 82.05) | 10.64 (7.72, 13.56) | 1.15 (1.11, 1.20) | 76.42 (74.66, 78.17) | 7.05 (4.32, 9.79) | 1.10 (1.06, 1.14) |
|  | 2015 | 74.92 (72.46, 77.37) | 84.03 (81.62, 86.45) | 9.12 (5.67, 12.57) | 1.12 (1.07, 1.17) | 78.41 (76.13, 80.69) | 3.49 (0.12, 6.86) | 1.05 (1.00, 1.09) |
|  | 2016 | 72.99 (71.05, 74.93) | 83.58 (81.44, 85.73) | 10.60 (7.70, 13.49) | 1.15 (1.10, 1.19) | 77.27 (75.39, 79.15) | 4.28 (1.58, 6.99) | 1.06 (1.02, 1.10) |
|  | 2017 | 74.06 (72.14, 75.99) | 85.24 (83.25, 87.22) | 11.18 (8.40, 13.95) | 1.15 (1.11, 1.19) | 79.96 (78.27, 81.65) | 5.90 (3.34, 8.46) | 1.08 (1.04, 1.12) |
|  | 2018 | 76.04 (74.10, 77.98) | 88.69 (87.12, 90.26) | 12.65 (10.15, 15.15) | 1.17 (1.13, 1.20) | 82.61 (81.01, 84.22) | 6.57 (4.05, 9.09) | 1.09 (1.05, 1.12) |
|  | 2019 | 76.57 (74.51, 78.63) | 88.28 (86.41, 90.15) | 11.71 (8.92, 14.50) | 1.15 (1.11, 1.19) | 83.72 (81.99, 85.44) | 7.15 (4.46, 9.84) | 1.09 (1.06, 1.13) |
| Daily sugar- sweetened beverage consumption | 2004 | - | - | - | - | - | - | - |
|  | 2005 | - | - | - | - | - | - | - |
|  | 2006 | 24.68 (22.36, 27.00) | 33.26 (30.51, 36.01) | 8.58 (4.98, 12.19) | 1.35 (1.18, 1.52) | 32.47 (30.55, 34.38) | 7.79 (4.78, 10.79) | 1.32 (1.17, 1.46) |
|  | 2007 | 21.58 (19.47, 23.69) | 31.80 (29.65, 33.96) | 10.22 (7.08, 13.37) | 1.47 (1.29, 1.66) | 29.46 (27.57, 31.35) | 7.88 (5.08, 10.69) | 1.37 (1.21, 1.52) |
|  | 2008 | 19.92 (17.68, 22.15) | 32.48 (30.20, 34.77) | 12.56 (9.48, 15.65) | 1.63 (1.42, 1.84) | 30.18 (28.21, 32.14) | 10.26 (7.33, 13.19) | 1.52 (1.32, 1.71) |
|  | 2009 | 22.66 (20.41, 24.90) | 34.72 (32.46, 36.98) | 12.06 (8.84, 15.29) | 1.53 (1.35, 1.72) | 32.47 (30.33, 34.62) | 9.82 (6.69, 12.94) | 1.43 (1.26, 1.61) |
|  | 2010 | 20.45 (17.72, 23.18) | 30.73 (28.02, 33.44) | 10.28 (6.37, 14.19) | 1.50 (1.26, 1.75) | 30.04 (27.79, 32.29) | 9.59 (6.12, 13.07) | 1.47 (1.25, 1.69) |
|  | 2011 | - | - | - | - | - | - | - |
|  | 2012 | 17.63 (15.47, 19.79) | 31.36 (27.84, 34.88) | 13.73 (9.59, 17.87) | 1.78 (1.48, 2.08) | 23.29 (21.26, 25.31) | 5.66 (2.70, 8.62) | 1.32 (1.12, 1.52) |
|  | 2013 | - | - | - | - | - | - | - |
|  | 2014 | 17.18 (15.45, 18.90) | 31.11 (28.28, 33.95) | 13.94 (10.61, 17.26) | 1.81 (1.57, 2.06) | 23.87 (21.96, 25.78) | 6.69 (4.12, 9.27) | 1.39 (1.21, 1.57) |
|  | 2015 | 13.86 (11.89, 15.82) | 28.15 (24.53, 31.77) | 14.29 (10.15, 18.44) | 2.03 (1.64, 2.42) | 22.29 (20.06, 24.52) | 8.43 (5.43, 11.44) | 1.61 (1.33, 1.89) |
|  | 2016 | 14.61 (13.03, 16.18) | 30.95 (28.01, 33.89) | 16.34 (13.00, 19.68) | 2.12 (1.81, 2.42) | 22.78 (20.87, 24.70) | 8.18 (5.69, 10.66) | 1.56 (1.35, 1.77) |
|  | 2017 | 14.70 (12.99, 16.41) | 30.42 (27.27, 33.57) | 15.72 (12.14, 19.31) | 2.07 (1.75, 2.39) | 23.50 (21.71, 25.30) | 8.80 (6.31, 11.30) | 1.60 (1.38, 1.82) |
|  | 2018 | 13.97 (12.34, 15.60) | 31.81 (28.73, 34.89) | 17.84 (14.35, 21.32) | 2.28 (1.93, 2.62) | 24.36 (22.51, 26.22) | 10.39 (7.92, 12.86) | 1.74 (1.50, 1.99) |
|  | 2019 | 13.01 (11.21, 14.81) | 32.08 (28.40, 35.75) | 19.07 (14.97, 23.16) | 2.47 (2.02, 2.91) | 25.02 (22.98, 27.07) | 12.01 (9.29, 14.74) | 1.92 (1.61, 2.23) |
| High total lifestyle risk^a^ | 2004 | 42.96 (40.19, 45.73) | 54.69 (52.27, 57.10) | 11.73 (8.05, 15.41) | 1.27 (1.17, 1.37) | 53.13 (51.10, 55.15) | 10.17 (6.73, 13.60) | 1.24 (1.14, 1.33) |
|  | 2005 | 38.25 (35.97, 40.52) | 52.99 (50.77, 55.20) | 14.74 (11.55, 17.93) | 1.39 (1.28, 1.49) | 51.16 (49.40, 52.92) | 12.91 (10.03, 15.80) | 1.34 (1.25, 1.43) |
|  | 2006 | 35.20 (32.62, 37.78) | 51.76 (49.01, 54.51) | 16.56 (12.78, 20.33) | 1.47 (1.34, 1.60) | 49.00 (46.89, 51.11) | 13.80 (10.46, 17.13) | 1.39 (1.27, 1.51) |
|  | 2007 | 37.11 (34.49, 39.74) | 49.11 (46.69, 51.52) | 11.99 (8.32, 15.67) | 1.32 (1.21, 1.44) | 45.30 (43.14, 47.47) | 8.19 (4.79, 11.59) | 1.22 (1.12, 1.33) |
|  | 2008 | 37.58 (34.85, 40.30) | 49.93 (47.63, 52.23) | 12.36 (8.76, 15.95) | 1.33 (1.21, 1.44) | 45.41 (43.14, 47.67) | 7.83 (4.21, 11.45) | 1.21 (1.10, 1.32) |
|  | 2009 | 35.68 (33.32, 38.03) | 49.50 (47.38, 51.62) | 13.83 (10.65, 17.00) | 1.39 (1.28, 1.50) | 45.80 (43.68, 47.92) | 10.12 (6.96, 13.29) | 1.28 (1.18, 1.39) |
|  | 2010 | 35.71 (33.01, 38.40) | 48.47 (46.04, 50.90) | 12.76 (9.14, 16.39) | 1.36 (1.23, 1.48) | 45.14 (42.93, 47.36) | 9.44 (5.94, 12.94) | 1.26 (1.15, 1.38) |
|  | 2011 | 36.49 (33.92, 39.07) | 50.35 (47.90, 52.81) | 13.86 (10.31, 17.41) | 1.38 (1.26, 1.50) | 45.19 (42.83, 47.56) | 8.70 (5.19, 12.21) | 1.24 (1.13, 1.35) |
|  | 2012 | 36.23 (32.81, 39.66) | 54.13 (49.60, 58.66) | 17.90 (12.33, 23.46) | 1.49 (1.31, 1.68) | 47.78 (44.27, 51.30) | 11.55 (6.40, 16.70) | 1.32 (1.15, 1.48) |
|  | 2013 | 35.85 (33.72, 37.97) | 51.44 (48.77, 54.11) | 15.60 (12.18, 19.01) | 1.44 (1.32, 1.55) | 46.18 (44.16, 48.20) | 10.33 (7.39, 13.28) | 1.29 (1.19, 1.38) |
|  | 2014 | 34.07 (31.89, 36.24) | 48.30 (45.48, 51.12) | 14.23 (10.67, 17.80) | 1.42 (1.29, 1.54) | 46.36 (44.12, 48.60) | 12.30 (9.18, 15.41) | 1.36 (1.25, 1.47) |
|  | 2015 | 34.20 (31.33, 37.07) | 50.49 (45.46, 55.52) | 16.29 (10.48, 22.10) | 1.48 (1.28, 1.67) | 44.57 (41.70, 47.43) | 10.37 (6.29, 14.44) | 1.30 (1.16, 1.44) |
|  | 2016 | 34.29 (32.23, 36.35) | 53.89 (50.90, 56.88) | 19.60 (15.96, 23.25) | 1.57 (1.44, 1.70) | 44.86 (42.60, 47.12) | 10.57 (7.52, 13.63) | 1.31 (1.21, 1.41) |
|  | 2017 | 33.73 (31.58, 35.87) | 54.71 (51.68, 57.74) | 20.98 (17.27, 24.69) | 1.62 (1.49, 1.76) | 47.77 (45.66, 49.88) | 14.05 (11.01, 17.08) | 1.42 (1.31, 1.53) |
|  | 2018 | 34.63 (32.45, 36.81) | 55.67 (52.66, 58.68) | 21.04 (17.32, 24.76) | 1.61 (1.47, 1.74) | 47.91 (45.72, 50.09) | 13.28 (10.17, 16.39) | 1.38 (1.28, 1.49) |
|  | 2019 | 34.01 (31.59, 36.43) | 55.99 (52.41, 59.57) | 21.98 (17.69, 26.27) | 1.65 (1.49, 1.80) | 48.18 (45.81, 50.55) | 14.17 (10.78, 17.56) | 1.42 (1.29, 1.54) |
| High total lifestyle risk (supplementary analysis)^b^ | 2004 | - | - | - | - | - | - | - |
|  | 2005 | - | - | - | - | - | - | - |
|  | 2006 | 45.75 (43.05, 48.45) | 64.18 (61.63, 66.72) | 18.42 (14.70, 22.15) | 1.40 (1.30, 1.50) | 61.57 (59.59, 63.55) | 15.82 (12.46, 19.18) | 1.35 (1.26, 1.44) |
|  | 2007 | 47.07 (44.42, 49.73) | 61.19 (58.98, 63.39) | 14.11 (10.60, 17.63) | 1.30 (1.21, 1.39) | 57.77 (55.54, 60.00) | 10.70 (7.25, 14.14) | 1.23 (1.14, 1.31) |
|  | 2008 | 45.65 (42.86, 48.45) | 61.25 (59.06, 63.44) | 15.59 (12.03, 19.16) | 1.34 (1.25, 1.44) | 57.04 (54.83, 59.25) | 11.39 (7.88, 14.90) | 1.25 (1.16, 1.34) |
|  | 2009 | 46.69 (44.13, 49.25) | 61.39 (59.33, 63.46) | 14.70 (11.41, 17.99) | 1.31 (1.23, 1.40) | 58.78 (56.66, 60.91) | 12.09 (8.75, 15.43) | 1.26 (1.18, 1.34) |
|  | 2010 | 44.27 (41.36, 47.17) | 60.07 (57.59, 62.55) | 15.81 (11.94, 19.67) | 1.36 (1.25, 1.46) | 57.69 (55.40, 59.99) | 13.43 (9.72, 17.13) | 1.30 (1.20, 1.40) |
|  | 2011 | - | - | - | - | - | - | - |
|  | 2012 | 44.35 (40.91, 47.78) | 64.62 (60.56, 68.67) | 20.27 (15.03, 25.50) | 1.46 (1.31, 1.60) | 56.31 (52.80, 59.82) | 11.96 (6.88, 17.05) | 1.27 (1.14, 1.40) |
|  | 2013 | - | - | - | - | - | - | - |
|  | 2014 | 42.06 (39.81, 44.31) | 60.33 (57.59, 63.07) | 18.27 (14.71, 21.83) | 1.43 (1.33, 1.54) | 54.40 (52.22, 56.58) | 12.34 (9.21, 15.48) | 1.29 (1.21, 1.38) |
|  | 2015 | 40.35 (37.33, 43.37) | 59.67 (54.46, 64.87) | 19.32 (13.28, 25.37) | 1.48 (1.31, 1.65) | 52.78 (49.89, 55.66) | 12.43 (8.23, 16.63) | 1.31 (1.19, 1.43) |
|  | 2016 | 40.99 (38.85, 43.14) | 64.72 (61.92, 67.51) | 23.72 (20.18, 27.27) | 1.58 (1.47, 1.69) | 54.45 (52.19, 56.70) | 13.46 (10.34, 16.57) | 1.33 (1.24, 1.42) |
|  | 2017 | 41.02 (38.78, 43.26) | 64.53 (61.73, 67.34) | 23.52 (19.93, 27.10) | 1.57 (1.46, 1.68) | 56.66 (54.59, 58.74) | 15.65 (12.57, 18.72) | 1.38 (1.29, 1.47) |
|  | 2018 | 41.37 (39.11, 43.62) | 66.21 (63.41, 69.02) | 24.85 (21.24, 28.46) | 1.60 (1.49, 1.71) | 57.00 (54.85, 59.14) | 15.63 (12.51, 18.76) | 1.38 (1.29, 1.47) |
|  | 2019 | 39.79 (37.28, 42.30) | 66.33 (63.09, 69.57) | 26.54 (22.48, 30.60) | 1.67 (1.54, 1.80) | 58.44 (56.09, 60.78) | 18.65 (15.20, 22.09) | 1.47 (1.36, 1.58) |

Abbreviations: CI, confidence interval.

* Risk differences and risk ratios are compared with base category of 'university' education.

^a^ Defined as engaging in two or more lifestyle risk behaviours, based on the following four individual risk behaviours: excessive alcohol consumption, insufficient physical activity, insufficient fruit and/or vegetable consumption, and current smoking.

^b^ Defined as engaging in two or more lifestyle risk behaviours, based on the following five individual risk behaviours: excessive alcohol consumption, insufficient physical activity, insufficient fruit and/or vegetable consumption, current smoking, and daily sugar-sweetened beverage consumption.
