# Supplementary material for: Sixteen-year trends in multiple lifestyle risk behaviours by socioeconomic status from 2004 to 2019 in New South Wales, Australia
Source: PLOS Glob Public Health. 2023 Feb 15;3(2):e0001606. doi: 10.1371/journal.pgph.0001606 (PMC10021655; doi:10.1371/journal.pgph.0001606)
Supplement: S4 Table — (DOCX) [file pgph.0001606.s008.docx]

**S4 Table. Prevalence, prevalence differences and prevalence ratios of individual lifestyle risk factors and combined lifestyle risk index by area-level disadvantage (IRSD), by year, persons 16 years and over, 2004-2019, NSW, Australia.**

| **Lifestyle risk factor** | **Year** | **Least disadvantaged** | **Middle disadvantage** | | | **Most disadvantaged** | | |
| --- | --- | --- | --- | --- | --- | --- | --- | --- |
|  |  | **Prevalence %  (95% CI)** | **Prevalence %  (95% CI)** | **Risk difference*  (95% CI)** | **Relative risk*  (95% CI)** | **Prevalence %  (95% CI)** | **Risk difference*  (95% CI)** | **Relative risk*  (95% CI)** |
| Current smoking | 2004 | 17.81 (15.10, 20.52) | 22.12 (20.73, 23.51) | 4.31 (1.27, 7.35) | 1.24 (1.04, 1.45) | 25.36 (22.86, 27.87) | 7.55 (3.86, 11.24) | 1.42 (1.17, 1.68) |
|  | 2005 | 15.34 (13.23, 17.44) | 20.29 (19.07, 21.51) | 4.95 (2.52, 7.39) | 1.32 (1.12, 1.52) | 26.61 (24.24, 28.98) | 11.27 (8.10, 14.44) | 1.74 (1.45, 2.02) |
|  | 2006 | 13.41 (11.02, 15.81) | 17.55 (16.17, 18.93) | 4.14 (1.37, 6.91) | 1.31 (1.05, 1.56) | 23.54 (20.82, 26.25) | 10.12 (6.50, 13.75) | 1.75 (1.38, 2.13) |
|  | 2007 | 12.38 (10.06, 14.71) | 17.45 (16.19, 18.71) | 5.07 (2.46, 7.68) | 1.41 (1.13, 1.70) | 22.75 (20.37, 25.14) | 10.37 (7.13, 13.61) | 1.84 (1.45, 2.23) |
|  | 2008 | 13.58 (11.17, 15.98) | 18.58 (17.20, 19.95) | 5.00 (2.23, 7.76) | 1.37 (1.11, 1.63) | 23.14 (20.44, 25.84) | 9.56 (5.92, 13.20) | 1.71 (1.34, 2.07) |
|  | 2009 | 12.11 (9.99, 14.24) | 18.23 (16.92, 19.54) | 6.12 (3.62, 8.61) | 1.51 (1.22, 1.79) | 21.53 (19.05, 24.01) | 9.42 (6.14, 12.69) | 1.78 (1.40, 2.15) |
|  | 2010 | 11.73 (9.54, 13.92) | 16.22 (14.89, 17.55) | 4.50 (1.94, 7.06) | 1.38 (1.10, 1.67) | 22.55 (19.91, 25.19) | 10.83 (7.40, 14.25) | 1.92 (1.50, 2.35) |
|  | 2011 | 8.48 (6.48, 10.47) | 15.23 (13.83, 16.62) | 6.75 (4.32, 9.18) | 1.80 (1.34, 2.25) | 19.90 (17.24, 22.55) | 11.42 (8.10, 14.74) | 2.35 (1.71, 2.98) |
|  | 2012 | 9.59 (7.81, 11.38) | 18.70 (16.70, 20.71) | 9.11 (6.42, 11.80) | 1.95 (1.53, 2.37) | 20.27 (16.60, 23.94) | 10.67 (6.59, 14.76) | 2.11 (1.56, 2.66) |
|  | 2013 | 11.73 (9.64, 13.83) | 16.86 (15.59, 18.14) | 5.13 (2.68, 7.58) | 1.44 (1.16, 1.72) | 21.37 (18.75, 23.99) | 9.64 (6.28, 13.00) | 1.82 (1.43, 2.22) |
|  | 2014 | 10.70 (8.70, 12.70) | 15.83 (14.53, 17.13) | 5.13 (2.75, 7.51) | 1.48 (1.18, 1.78) | 23.94 (21.15, 26.74) | 13.24 (9.81, 16.68) | 2.24 (1.75, 2.73) |
|  | 2015 | 10.77 (7.33, 14.21) | 14.45 (12.94, 15.95) | 3.68 (-0.08, 7.44) | 1.34 (0.89, 1.79) | 17.38 (14.37, 20.40) | 6.62 (2.05, 11.18) | 1.61 (1.03, 2.20) |
|  | 2016 | 11.26 (9.31, 13.22) | 16.19 (14.78, 17.60) | 4.93 (2.52, 7.34) | 1.44 (1.16, 1.72) | 19.94 (17.25, 22.62) | 8.67 (5.35, 11.99) | 1.77 (1.38, 2.16) |
|  | 2017 | 11.45 (9.36, 13.53) | 16.42 (15.05, 17.79) | 4.97 (2.48, 7.46) | 1.43 (1.15, 1.72) | 20.85 (18.07, 23.62) | 9.40 (5.93, 12.86) | 1.82 (1.41, 2.23) |
|  | 2018 | 10.45 (8.55, 12.36) | 16.31 (14.92, 17.71) | 5.86 (3.50, 8.22) | 1.56 (1.25, 1.87) | 21.00 (18.13, 23.88) | 10.55 (7.10, 14.00) | 2.01 (1.55, 2.47) |
|  | 2019 | 9.30 (7.31, 11.29) | 17.30 (15.73, 18.88) | 8.00 (5.47, 10.54) | 1.86 (1.43, 2.29) | 26.07 (22.52, 29.62) | 16.77 (12.70, 20.84) | 2.80 (2.09, 3.51) |
| Excessive alcohol consumption | 2004 | 18.25 (15.68, 20.82) | 14.85 (13.73, 15.98) | -3.40 (-6.20, -0.59) | 0.81 (0.68, 0.94) | 13.59 (11.83, 15.34) | -4.66 (-7.77, -1.56) | 0.74 (0.60, 0.89) |
|  | 2005 | 16.38 (14.41, 18.36) | 13.62 (12.64, 14.60) | -2.77 (-4.97, -0.56) | 0.83 (0.71, 0.95) | 13.21 (11.55, 14.87) | -3.18 (-5.75, -0.60) | 0.81 (0.67, 0.95) |
|  | 2006 | 17.45 (14.93, 19.97) | 15.37 (14.15, 16.59) | -2.08 (-4.89, 0.73) | 0.88 (0.74, 1.03) | 12.68 (10.81, 14.56) | -4.77 (-7.92, -1.62) | 0.73 (0.58, 0.88) |
|  | 2007 | 16.75 (14.31, 19.18) | 13.70 (12.54, 14.86) | -3.04 (-5.71, -0.38) | 0.82 (0.68, 0.96) | 11.62 (9.89, 13.36) | -5.12 (-8.15, -2.10) | 0.70 (0.55, 0.84) |
|  | 2008 | 18.17 (15.64, 20.71) | 15.06 (13.89, 16.23) | -3.11 (-5.88, -0.34) | 0.83 (0.70, 0.96) | 14.21 (12.14, 16.27) | -3.97 (-7.23, -0.70) | 0.78 (0.63, 0.94) |
|  | 2009 | 17.95 (15.72, 20.19) | 15.94 (14.86, 17.02) | -2.01 (-4.50, 0.47) | 0.89 (0.76, 1.01) | 13.97 (12.12, 15.81) | -3.99 (-6.89, -1.09) | 0.78 (0.64, 0.92) |
|  | 2010 | 16.13 (13.70, 18.57) | 13.63 (12.54, 14.73) | -2.50 (-5.17, 0.17) | 0.84 (0.70, 0.99) | 12.02 (10.11, 13.93) | -4.12 (-7.21, -1.02) | 0.74 (0.58, 0.91) |
|  | 2011 | 16.02 (13.46, 18.59) | 14.10 (12.91, 15.29) | -1.92 (-4.75, 0.91) | 0.88 (0.72, 1.04) | 12.22 (10.26, 14.18) | -3.80 (-7.03, -0.57) | 0.76 (0.59, 0.94) |
|  | 2012 | 15.97 (13.46, 18.48) | 12.90 (11.50, 14.31) | -3.07 (-5.95, -0.19) | 0.81 (0.65, 0.96) | 12.37 (9.12, 15.62) | -3.60 (-7.71, 0.50) | 0.77 (0.54, 1.01) |
|  | 2013 | 13.22 (11.12, 15.32) | 12.47 (11.43, 13.50) | -0.76 (-3.10, 1.59) | 0.94 (0.77, 1.11) | 11.05 (9.09, 13.01) | -2.17 (-5.05, 0.70) | 0.84 (0.64, 1.03) |
|  | 2014 | 15.97 (13.72, 18.23) | 12.92 (11.79, 14.05) | -3.06 (-5.58, -0.53) | 0.81 (0.67, 0.94) | 10.93 (9.03, 12.83) | -5.05 (-7.99, -2.10) | 0.68 (0.53, 0.84) |
|  | 2015 | 13.33 (10.53, 16.14) | 12.81 (11.32, 14.31) | -0.52 (-3.70, 2.67) | 0.96 (0.73, 1.19) | 9.74 (7.92, 11.56) | -3.59 (-6.94, -0.24) | 0.73 (0.52, 0.94) |
|  | 2016 | 16.09 (13.95, 18.23) | 13.59 (12.39, 14.79) | -2.50 (-4.95, -0.05) | 0.84 (0.71, 0.98) | 11.27 (9.36, 13.17) | -4.82 (-7.68, -1.96) | 0.70 (0.55, 0.85) |
|  | 2017 | 15.78 (13.64, 17.93) | 13.64 (12.52, 14.76) | -2.14 (-4.56, 0.28) | 0.86 (0.73, 1.00) | 13.11 (10.94, 15.27) | -2.68 (-5.72, 0.36) | 0.83 (0.65, 1.01) |
|  | 2018 | 16.78 (14.62, 18.94) | 14.08 (12.89, 15.28) | -2.70 (-5.16, -0.23) | 0.84 (0.71, 0.97) | 13.20 (10.96, 15.44) | -3.58 (-6.69, -0.47) | 0.79 (0.62, 0.95) |
|  | 2019 | 16.67 (14.24, 19.09) | 12.93 (11.75, 14.10) | -3.74 (-6.44, -1.04) | 0.78 (0.64, 0.91) | 12.63 (10.33, 14.92) | -4.04 (-7.38, -0.70) | 0.76 (0.58, 0.93) |
| Insufficient physical activity | 2004 | 35.32 (32.00, 38.64) | 38.98 (37.42, 40.55) | 3.66 (0.00, 7.33) | 1.10 (0.99, 1.22) | 43.43 (40.67, 46.20) | 8.11 (3.79, 12.43) | 1.23 (1.09, 1.37) |
|  | 2005 | 32.60 (30.02, 35.17) | 39.08 (37.64, 40.51) | 6.48 (3.53, 9.43) | 1.20 (1.09, 1.30) | 43.37 (40.84, 45.90) | 10.78 (7.17, 14.38) | 1.33 (1.20, 1.46) |
|  | 2006 | 30.98 (27.94, 34.03) | 36.97 (35.31, 38.63) | 5.99 (2.51, 9.47) | 1.19 (1.06, 1.32) | 43.30 (40.15, 46.44) | 12.32 (7.94, 16.69) | 1.40 (1.23, 1.57) |
|  | 2007 | 31.92 (28.85, 35.00) | 36.98 (35.35, 38.61) | 5.05 (1.66, 8.44) | 1.16 (1.04, 1.28) | 39.81 (36.60, 43.02) | 7.89 (3.47, 12.31) | 1.25 (1.09, 1.40) |
|  | 2008 | 29.20 (26.11, 32.30) | 37.28 (35.72, 38.85) | 8.08 (4.60, 11.56) | 1.28 (1.13, 1.42) | 40.47 (37.39, 43.55) | 11.27 (7.04, 15.49) | 1.39 (1.21, 1.56) |
|  | 2009 | 28.19 (25.67, 30.72) | 36.14 (34.60, 37.68) | 7.95 (5.00, 10.91) | 1.28 (1.16, 1.41) | 39.41 (36.72, 42.11) | 11.22 (7.51, 14.93) | 1.40 (1.24, 1.56) |
|  | 2010 | 31.56 (28.42, 34.71) | 36.23 (34.56, 37.90) | 4.67 (1.12, 8.21) | 1.15 (1.02, 1.27) | 39.51 (36.66, 42.36) | 7.95 (3.72, 12.17) | 1.25 (1.10, 1.41) |
|  | 2011 | 32.09 (28.71, 35.46) | 37.40 (35.70, 39.11) | 5.31 (1.52, 9.11) | 1.17 (1.03, 1.30) | 40.80 (37.86, 43.73) | 8.71 (4.24, 13.17) | 1.27 (1.11, 1.43) |
|  | 2012 | 34.18 (30.33, 38.04) | 40.21 (37.64, 42.77) | 6.02 (1.38, 10.67) | 1.18 (1.02, 1.33) | 44.60 (38.33, 50.88) | 10.42 (3.07, 17.77) | 1.31 (1.07, 1.54) |
|  | 2013 | 30.56 (27.94, 33.18) | 37.60 (36.08, 39.12) | 7.04 (4.02, 10.07) | 1.23 (1.11, 1.35) | 43.48 (40.61, 46.35) | 12.92 (9.05, 16.80) | 1.42 (1.27, 1.58) |
|  | 2014 | 28.68 (25.83, 31.53) | 33.77 (32.12, 35.41) | 5.09 (1.80, 8.37) | 1.18 (1.05, 1.31) | 37.10 (34.21, 40.00) | 8.43 (4.37, 12.48) | 1.29 (1.13, 1.46) |
|  | 2015 | 24.14 (20.37, 27.91) | 33.96 (31.96, 35.97) | 9.83 (5.56, 14.09) | 1.41 (1.17, 1.64) | 39.81 (35.64, 43.97) | 15.67 (10.01, 21.32) | 1.65 (1.34, 1.96) |
|  | 2016 | 24.96 (22.47, 27.45) | 34.21 (32.57, 35.86) | 9.26 (6.25, 12.26) | 1.37 (1.22, 1.52) | 39.62 (36.64, 42.59) | 14.66 (10.78, 18.54) | 1.59 (1.39, 1.79) |
|  | 2017 | 24.00 (21.56, 26.43) | 35.27 (33.63, 36.90) | 11.27 (8.33, 14.21) | 1.47 (1.31, 1.63) | 38.49 (35.48, 41.49) | 14.49 (10.62, 18.36) | 1.60 (1.40, 1.81) |
|  | 2018 | 21.23 (18.97, 23.49) | 33.47 (31.79, 35.14) | 12.24 (9.42, 15.06) | 1.58 (1.39, 1.76) | 38.45 (35.27, 41.62) | 17.22 (13.33, 21.10) | 1.81 (1.57, 2.05) |
|  | 2019 | 24.68 (21.90, 27.47) | 31.19 (29.41, 32.96) | 6.50 (3.20, 9.81) | 1.26 (1.10, 1.42) | 37.03 (33.45, 40.61) | 12.35 (7.78, 16.92) | 1.50 (1.28, 1.73) |
| Insufficient fruit and/or vegetable consumption | 2004 | 77.61 (74.85, 80.38) | 78.60 (77.32, 79.87) | 0.98 (-2.06, 4.03) | 1.01 (0.97, 1.05) | 81.95 (79.92, 83.98) | 4.33 (0.91, 7.76) | 1.06 (1.01, 1.10) |
|  | 2005 | 75.76 (73.56, 77.97) | 75.48 (74.31, 76.65) | -0.28 (-2.78, 2.22) | 1.00 (0.96, 1.03) | 76.99 (74.98, 78.99) | 1.22 (-1.76, 4.21) | 1.02 (0.98, 1.06) |
|  | 2006 | 72.21 (69.41, 75.00) | 73.46 (71.98, 74.93) | 1.25 (-1.92, 4.43) | 1.02 (0.97, 1.06) | 75.75 (73.04, 78.45) | 3.54 (-0.37, 7.45) | 1.05 (0.99, 1.10) |
|  | 2007 | 69.33 (66.38, 72.28) | 73.27 (71.91, 74.64) | 3.94 (0.80, 7.09) | 1.06 (1.01, 1.10) | 76.22 (74.08, 78.37) | 6.89 (3.30, 10.49) | 1.10 (1.04, 1.15) |
|  | 2008 | 69.65 (66.85, 72.44) | 72.30 (70.83, 73.78) | 2.65 (-0.52, 5.83) | 1.04 (0.99, 1.09) | 76.83 (74.46, 79.21) | 7.18 (3.60, 10.76) | 1.10 (1.05, 1.16) |
|  | 2009 | 68.63 (65.92, 71.33) | 70.85 (69.46, 72.23) | 2.22 (-0.82, 5.26) | 1.03 (0.99, 1.08) | 73.69 (71.09, 76.29) | 5.06 (1.31, 8.81) | 1.07 (1.02, 1.13) |
|  | 2010 | 69.29 (66.21, 72.38) | 72.78 (71.33, 74.23) | 3.49 (0.08, 6.90) | 1.05 (1.00, 1.10) | 75.26 (72.74, 77.77) | 5.96 (1.98, 9.95) | 1.09 (1.03, 1.15) |
|  | 2011 | 68.82 (65.79, 71.85) | 74.84 (73.37, 76.31) | 6.02 (2.65, 9.38) | 1.09 (1.04, 1.14) | 74.95 (72.27, 77.63) | 6.13 (2.08, 10.17) | 1.09 (1.03, 1.15) |
|  | 2012 | 71.69 (68.58, 74.80) | 72.84 (70.62, 75.06) | 1.14 (-2.68, 4.97) | 1.02 (0.96, 1.07) | 78.42 (74.38, 82.47) | 6.73 (1.64, 11.83) | 1.09 (1.02, 1.17) |
|  | 2013 | 71.25 (68.54, 73.96) | 73.92 (72.55, 75.30) | 2.67 (-0.37, 5.71) | 1.04 (0.99, 1.08) | 77.54 (75.18, 79.91) | 6.29 (2.70, 9.88) | 1.09 (1.04, 1.14) |
|  | 2014 | 72.82 (70.04, 75.61) | 74.18 (72.71, 75.65) | 1.35 (-1.80, 4.51) | 1.02 (0.97, 1.06) | 78.58 (76.23, 80.93) | 5.76 (2.13, 9.39) | 1.08 (1.03, 1.13) |
|  | 2015 | 77.32 (74.24, 80.40) | 78.60 (76.90, 80.30) | 1.27 (-2.24, 4.79) | 1.02 (0.97, 1.06) | 79.31 (75.51, 83.12) | 1.99 (-2.91, 6.89) | 1.03 (0.96, 1.09) |
|  | 2016 | 72.12 (69.46, 74.78) | 77.55 (76.05, 79.04) | 5.42 (2.37, 8.47) | 1.08 (1.03, 1.12) | 80.80 (78.30, 83.30) | 8.68 (5.02, 12.33) | 1.12 (1.07, 1.17) |
|  | 2017 | 73.78 (71.15, 76.40) | 79.58 (78.22, 80.95) | 5.81 (2.86, 8.76) | 1.08 (1.04, 1.12) | 82.25 (79.68, 84.82) | 8.48 (4.81, 12.14) | 1.11 (1.06, 1.17) |
|  | 2018 | 77.16 (74.62, 79.70) | 81.41 (80.05, 82.77) | 4.25 (1.37, 7.12) | 1.06 (1.02, 1.09) | 85.47 (83.19, 87.74) | 8.31 (4.89, 11.72) | 1.11 (1.06, 1.15) |
|  | 2019 | 76.69 (73.88, 79.50) | 82.32 (80.88, 83.75) | 5.62 (2.47, 8.78) | 1.07 (1.03, 1.12) | 86.03 (83.44, 88.62) | 9.34 (5.52, 13.16) | 1.12 (1.07, 1.17) |
| Daily sugar -sweetened beverage consumption | 2004 | - | - | - | - | - | - | - |
|  | 2005 | - | - | - | - | - | - | - |
|  | 2006 | 22.24 (19.31, 25.16) | 31.01 (29.37, 32.66) | 8.77 (5.41, 12.13) | 1.39 (1.20, 1.59) | 34.51 (31.50, 37.52) | 12.27 (8.07, 16.48) | 1.55 (1.31, 1.80) |
|  | 2007 | 20.13 (17.48, 22.78) | 28.48 (27.00, 29.95) | 8.35 (5.44, 11.25) | 1.42 (1.22, 1.61) | 31.88 (29.23, 34.53) | 11.75 (7.91, 15.59) | 1.59 (1.33, 1.84) |
|  | 2008 | 21.41 (18.60, 24.22) | 28.30 (26.72, 29.88) | 6.89 (3.76, 10.03) | 1.32 (1.14, 1.51) | 31.93 (29.06, 34.80) | 10.52 (6.55, 14.49) | 1.49 (1.26, 1.73) |
|  | 2009 | 23.11 (20.15, 26.06) | 30.21 (28.54, 31.89) | 7.11 (3.65, 10.56) | 1.31 (1.12, 1.49) | 35.27 (32.15, 38.39) | 12.16 (7.94, 16.38) | 1.53 (1.29, 1.76) |
|  | 2010 | 22.20 (18.84, 25.56) | 26.86 (25.06, 28.66) | 4.65 (0.83, 8.48) | 1.21 (1.01, 1.41) | 32.29 (28.89, 35.69) | 10.08 (5.35, 14.81) | 1.46 (1.19, 1.72) |
|  | 2011 | - | - | - | - | - | - | - |
|  | 2012 | 18.21 (15.27, 21.15) | 24.43 (22.69, 26.16) | 6.22 (2.81, 9.63) | 1.34 (1.10, 1.58) | 25.01 (21.41, 28.62) | 6.81 (2.15, 11.46) | 1.37 (1.08, 1.67) |
|  | 2013 | - | - | - | - | - | - | - |
|  | 2014 | 15.43 (12.99, 17.87) | 23.67 (22.15, 25.20) | 8.24 (5.36, 11.12) | 1.53 (1.27, 1.80) | 28.34 (25.50, 31.18) | 12.90 (9.16, 16.64) | 1.84 (1.49, 2.18) |
|  | 2015 | 14.18 (11.32, 17.04) | 20.71 (18.95, 22.47) | 6.53 (3.17, 9.89) | 1.46 (1.14, 1.78) | 25.76 (22.34, 29.17) | 11.57 (7.12, 16.03) | 1.82 (1.38, 2.26) |
|  | 2016 | 15.28 (13.06, 17.51) | 21.86 (20.33, 23.39) | 6.58 (3.88, 9.28) | 1.43 (1.20, 1.66) | 24.45 (21.74, 27.16) | 9.17 (5.66, 12.67) | 1.60 (1.31, 1.89) |
|  | 2017 | 13.70 (11.60, 15.81) | 21.82 (20.34, 23.30) | 8.12 (5.54, 10.69) | 1.59 (1.32, 1.86) | 27.75 (24.67, 30.83) | 14.05 (10.31, 17.78) | 2.03 (1.64, 2.41) |
|  | 2018 | 11.83 (9.93, 13.73) | 22.79 (21.27, 24.30) | 10.96 (8.53, 13.39) | 1.93 (1.59, 2.26) | 27.91 (24.84, 30.98) | 16.08 (12.47, 19.69) | 2.36 (1.90, 2.82) |
|  | 2019 | 13.03 (10.73, 15.32) | 21.38 (19.74, 23.03) | 8.35 (5.53, 11.17) | 1.64 (1.33, 1.96) | 30.68 (27.05, 34.31) | 17.65 (13.36, 21.95) | 2.35 (1.86, 2.85) |
| High total lifestyle risk^a^ | 2004 | 47.71 (44.17, 51.25) | 49.72 (48.06, 51.39) | 2.01 (-1.90, 5.92) | 1.04 (0.96, 1.13) | 55.49 (52.63, 58.34) | 7.78 (3.23, 12.32) | 1.16 (1.06, 1.27) |
|  | 2005 | 43.22 (40.43, 46.00) | 47.33 (45.83, 48.84) | 4.12 (0.95, 7.28) | 1.10 (1.02, 1.17) | 53.35 (50.70, 55.99) | 10.13 (6.28, 13.97) | 1.23 (1.13, 1.33) |
|  | 2006 | 40.63 (37.18, 44.08) | 44.84 (43.05, 46.62) | 4.20 (0.31, 8.10) | 1.10 (1.00, 1.21) | 50.59 (47.37, 53.80) | 9.95 (5.24, 14.67) | 1.25 (1.11, 1.38) |
|  | 2007 | 38.42 (35.06, 41.79) | 43.87 (42.17, 45.56) | 5.44 (1.73, 9.16) | 1.14 (1.03, 1.25) | 48.65 (45.56, 51.75) | 10.23 (5.84, 14.63) | 1.27 (1.14, 1.40) |
|  | 2008 | 39.33 (36.03, 42.62) | 44.23 (42.49, 45.97) | 4.91 (1.20, 8.61) | 1.12 (1.02, 1.23) | 49.57 (46.34, 52.80) | 10.24 (5.51, 14.98) | 1.26 (1.12, 1.40) |
|  | 2009 | 36.25 (33.28, 39.21) | 44.49 (42.86, 46.12) | 8.24 (4.86, 11.62) | 1.23 (1.12, 1.34) | 47.86 (44.90, 50.83) | 11.61 (7.41, 15.82) | 1.32 (1.18, 1.46) |
|  | 2010 | 38.67 (35.23, 42.12) | 42.53 (40.74, 44.32) | 3.86 (-0.02, 7.73) | 1.10 (0.99, 1.21) | 48.07 (45.04, 51.10) | 9.40 (4.82, 13.97) | 1.24 (1.11, 1.38) |
|  | 2011 | 37.12 (33.54, 40.69) | 44.56 (42.76, 46.37) | 7.45 (3.43, 11.46) | 1.20 (1.07, 1.33) | 47.33 (44.11, 50.54) | 10.21 (5.41, 15.02) | 1.28 (1.12, 1.43) |
|  | 2012 | 38.86 (35.04, 42.68) | 45.61 (42.96, 48.26) | 6.75 (2.03, 11.47) | 1.17 (1.04, 1.31) | 52.03 (45.89, 58.17) | 13.17 (5.98, 20.36) | 1.34 (1.13, 1.54) |
|  | 2013 | 36.47 (33.65, 39.29) | 44.13 (42.52, 45.75) | 7.67 (4.42, 10.91) | 1.21 (1.11, 1.31) | 50.92 (47.87, 53.97) | 14.45 (10.31, 18.59) | 1.40 (1.26, 1.53) |
|  | 2014 | 38.09 (34.97, 41.21) | 41.32 (39.57, 43.07) | 3.23 (-0.33, 6.79) | 1.08 (0.99, 1.18) | 49.28 (46.12, 52.43) | 11.19 (6.76, 15.61) | 1.29 (1.16, 1.43) |
|  | 2015 | 34.46 (29.59, 39.33) | 43.45 (41.23, 45.67) | 8.99 (3.61, 14.37) | 1.26 (1.07, 1.45) | 45.72 (41.42, 50.01) | 11.26 (4.75, 17.77) | 1.33 (1.10, 1.55) |
|  | 2016 | 34.42 (31.57, 37.27) | 42.95 (41.17, 44.72) | 8.53 (5.16, 11.89) | 1.25 (1.13, 1.36) | 49.87 (46.72, 53.01) | 15.45 (11.20, 19.69) | 1.45 (1.30, 1.60) |
|  | 2017 | 34.35 (31.50, 37.20) | 44.72 (42.98, 46.46) | 10.37 (7.03, 13.72) | 1.30 (1.18, 1.42) | 50.06 (46.81, 53.31) | 15.71 (11.39, 20.02) | 1.46 (1.30, 1.61) |
|  | 2018 | 34.43 (31.61, 37.26) | 44.79 (43.00, 46.58) | 10.36 (7.02, 13.69) | 1.30 (1.18, 1.42) | 51.90 (48.53, 55.28) | 17.47 (13.06, 21.88) | 1.51 (1.35, 1.67) |
|  | 2019 | 35.97 (32.72, 39.23) | 43.70 (41.74, 45.67) | 7.73 (3.91, 11.54) | 1.21 (1.09, 1.34) | 53.08 (49.23, 56.92) | 17.10 (12.10, 22.10) | 1.48 (1.31, 1.65) |
| High total lifestyle risk (supplementary analyses)^b^ | 2004 | - | - | - | - | - | - | - |
|  | 2005 | - | - | - | - | - | - | - |
|  | 2006 | 49.59 (46.16, 53.02) | 57.35 (55.61, 59.08) | 7.76 (3.90, 11.61) | 1.16 (1.07, 1.24) | 63.65 (60.56, 66.74) | 14.06 (9.45, 18.67) | 1.28 (1.18, 1.39) |
|  | 2007 | 47.72 (44.12, 51.31) | 56.06 (54.45, 57.67) | 8.34 (4.52, 12.17) | 1.18 (1.08, 1.27) | 60.53 (57.62, 63.43) | 12.81 (8.11, 17.51) | 1.27 (1.15, 1.38) |
|  | 2008 | 47.42 (44.07, 50.76) | 55.17 (53.38, 56.97) | 7.76 (3.95, 11.56) | 1.16 (1.07, 1.25) | 61.13 (58.14, 64.12) | 13.71 (9.21, 18.22) | 1.29 (1.18, 1.40) |
|  | 2009 | 47.39 (44.23, 50.54) | 56.60 (54.92, 58.27) | 9.21 (5.63, 12.79) | 1.19 (1.11, 1.28) | 60.70 (57.72, 63.67) | 13.31 (9.00, 17.62) | 1.28 (1.18, 1.39) |
|  | 2010 | 46.87 (43.21, 50.53) | 53.82 (51.98, 55.67) | 6.95 (2.88, 11.03) | 1.15 (1.05, 1.25) | 61.13 (57.99, 64.27) | 14.26 (9.41, 19.10) | 1.30 (1.18, 1.43) |
|  | 2011 | - | - | - | - | - | - | - |
|  | 2012 | 46.42 (42.47, 50.38) | 54.95 (52.41, 57.49) | 8.53 (3.82, 13.24) | 1.18 (1.07, 1.30) | 60.95 (54.85, 67.05) | 14.53 (7.19, 21.86) | 1.31 (1.14, 1.49) |
|  | 2013 | - | - | - | - | - | - | - |
|  | 2014 | 44.91 (41.71, 48.11) | 50.66 (48.92, 52.39) | 5.74 (2.11, 9.38) | 1.13 (1.04, 1.22) | 59.17 (56.10, 62.24) | 14.26 (9.84, 18.68) | 1.32 (1.20, 1.43) |
|  | 2015 | 40.82 (35.82, 45.82) | 51.26 (49.02, 53.51) | 10.44 (4.95, 15.94) | 1.26 (1.09, 1.42) | 54.25 (49.74, 58.76) | 13.43 (6.68, 20.18) | 1.33 (1.13, 1.53) |
|  | 2016 | 41.78 (38.81, 44.75) | 52.08 (50.30, 53.87) | 10.30 (6.83, 13.78) | 1.25 (1.15, 1.35) | 58.48 (55.39, 61.58) | 16.70 (12.42, 20.99) | 1.40 (1.28, 1.52) |
|  | 2017 | 40.60 (37.65, 43.55) | 53.29 (51.55, 55.03) | 12.69 (9.26, 16.12) | 1.31 (1.21, 1.42) | 60.65 (57.52, 63.79) | 20.06 (15.76, 24.35) | 1.49 (1.36, 1.63) |
|  | 2018 | 40.61 (37.67, 43.56) | 53.61 (51.81, 55.41) | 13.00 (9.55, 16.45) | 1.32 (1.21, 1.43) | 61.45 (58.15, 64.75) | 20.84 (16.42, 25.26) | 1.51 (1.38, 1.65) |
|  | 2019 | 41.16 (37.83, 44.49) | 52.57 (50.59, 54.55) | 11.41 (7.53, 15.29) | 1.28 (1.16, 1.39) | 64.11 (60.43, 67.79) | 22.95 (18.00, 27.90) | 1.56 (1.40, 1.71) |

Abbreviations: CI, confidence interval; IRSD, index of relative socioeconomic disadvantage.

* Risk differences and risk ratios are compared with the base category of 'least disadvantaged'.

^a^ Defined as engaging in two or more lifestyle risk behaviours, based on the following four individual risk behaviours: excessive alcohol consumption, insufficient physical activity, insufficient fruit and/or vegetable consumption, and current smoking.

^b^ Defined as engaging in two or more lifestyle risk behaviours, based on the following five individual risk behaviours: excessive alcohol consumption, insufficient physical activity, insufficient fruit and/or vegetable consumption, current smoking, and daily sugar-sweetened beverage consumption.
