# Supplementary material for: Sixteen-year trends in multiple lifestyle risk behaviours by socioeconomic status from 2004 to 2019 in New South Wales, Australia
Source: PLOS Glob Public Health. 2023 Feb 15;3(2):e0001606. doi: 10.1371/journal.pgph.0001606 (PMC10021655; doi:10.1371/journal.pgph.0001606)
Supplement: S5 Table — (DOCX) [file pgph.0001606.s009.docx]

**S5 Table. Prevalence, prevalence differences and prevalence ratios of individual lifestyle risk factors and combined lifestyle risk index by geographical remoteness (ARIA+), by year, persons 16 years and over, 2004-2019, NSW, Australia.**

| **Lifestyle risk factor** | **Year** | **Major city** | **Regional/remote** | | |
| --- | --- | --- | --- | --- | --- |
|  |  | **Prevalence %  (95% CI)** | **Prevalence %  (95% CI)** | **Risk difference*  (95% CI)** | **Relative risk*  (95% CI)** |
| Current smoking | 2004 | 21.38 (19.92, 22.84) | 23.12 (21.56, 24.67) | 1.74 (-0.39, 3.87) | 1.08 (0.98, 1.18) |
|  | 2005 | 20.01 (18.80, 21.23) | 21.84 (20.35, 23.34) | 1.83 (-0.09, 3.75) | 1.09 (0.99, 1.19) |
|  | 2006 | 16.87 (15.54, 18.21) | 20.73 (18.87, 22.59) | 3.85 (1.57, 6.14) | 1.23 (1.08, 1.38) |
|  | 2007 | 16.83 (15.57, 18.10) | 19.30 (17.63, 20.97) | 2.47 (0.39, 4.54) | 1.15 (1.02, 1.28) |
|  | 2008 | 18.04 (16.69, 19.39) | 19.50 (17.79, 21.21) | 1.46 (-0.72, 3.64) | 1.08 (0.96, 1.21) |
|  | 2009 | 17.17 (15.91, 18.42) | 18.87 (17.26, 20.47) | 1.70 (-0.34, 3.74) | 1.10 (0.98, 1.22) |
|  | 2010 | 15.43 (14.13, 16.73) | 19.70 (18.00, 21.41) | 4.28 (2.13, 6.42) | 1.28 (1.12, 1.43) |
|  | 2011 | 13.95 (12.64, 15.25) | 16.41 (14.70, 18.12) | 2.46 (0.31, 4.62) | 1.18 (1.01, 1.34) |
|  | 2012 | 16.44 (14.70, 18.17) | 19.46 (16.87, 22.05) | 3.02 (-0.09, 6.13) | 1.18 (0.98, 1.38) |
|  | 2013 | 15.65 (14.49, 16.81) | 19.33 (17.28, 21.38) | 3.68 (1.32, 6.03) | 1.23 (1.07, 1.39) |
|  | 2014 | 15.75 (14.54, 16.96) | 18.13 (16.06, 20.20) | 2.38 (-0.02, 4.79) | 1.15 (0.99, 1.31) |
|  | 2015 | 13.28 (11.72, 14.85) | 16.78 (14.66, 18.90) | 3.50 (0.85, 6.14) | 1.26 (1.04, 1.48) |
|  | 2016 | 14.94 (13.74, 16.14) | 18.78 (16.37, 21.18) | 3.84 (1.15, 6.53) | 1.26 (1.07, 1.45) |
|  | 2017 | 14.81 (13.63, 15.98) | 20.76 (18.33, 23.19) | 5.95 (3.25, 8.66) | 1.40 (1.20, 1.60) |
|  | 2018 | 15.29 (14.06, 16.52) | 17.68 (15.47, 19.88) | 2.39 (-0.14, 4.91) | 1.16 (0.98, 1.33) |
|  | 2019 | 16.32 (14.88, 17.76) | 19.76 (17.32, 22.20) | 3.44 (0.61, 6.27) | 1.21 (1.03, 1.39) |
| Excessive alcohol consumption | 2004 | 15.32 (14.05, 16.59) | 15.71 (14.52, 16.90) | 0.39 (-1.35, 2.12) | 1.03 (0.91, 1.14) |
|  | 2005 | 13.75 (12.75, 14.75) | 15.13 (13.89, 16.38) | 1.38 (-0.22, 2.98) | 1.10 (0.98, 1.22) |
|  | 2006 | 14.65 (13.43, 15.87) | 17.04 (15.49, 18.59) | 2.39 (0.41, 4.37) | 1.16 (1.02, 1.31) |
|  | 2007 | 13.60 (12.40, 14.80) | 14.82 (13.49, 16.15) | 1.22 (-0.57, 3.00) | 1.09 (0.95, 1.23) |
|  | 2008 | 15.34 (14.08, 16.60) | 16.12 (14.70, 17.53) | 0.78 (-1.12, 2.67) | 1.05 (0.92, 1.18) |
|  | 2009 | 15.65 (14.53, 16.77) | 16.99 (15.66, 18.32) | 1.34 (-0.40, 3.08) | 1.09 (0.97, 1.20) |
|  | 2010 | 13.36 (12.18, 14.54) | 15.00 (13.69, 16.30) | 1.64 (-0.12, 3.39) | 1.12 (0.98, 1.26) |
|  | 2011 | 13.69 (12.43, 14.95) | 15.44 (13.97, 16.91) | 1.75 (-0.19, 3.69) | 1.13 (0.98, 1.28) |
|  | 2012 | 12.89 (11.52, 14.25) | 14.69 (12.46, 16.92) | 1.80 (-0.82, 4.42) | 1.14 (0.93, 1.35) |
|  | 2013 | 11.29 (10.28, 12.30) | 15.05 (13.48, 16.62) | 3.76 (1.89, 5.63) | 1.33 (1.15, 1.52) |
|  | 2014 | 13.10 (11.99, 14.21) | 13.44 (11.91, 14.97) | 0.34 (-1.55, 2.23) | 1.03 (0.88, 1.17) |
|  | 2015 | 11.77 (10.33, 13.21) | 13.98 (12.25, 15.71) | 2.21 (-0.04, 4.46) | 1.19 (0.98, 1.39) |
|  | 2016 | 13.64 (12.52, 14.77) | 13.99 (12.34, 15.65) | 0.35 (-1.65, 2.35) | 1.03 (0.88, 1.17) |
|  | 2017 | 12.99 (11.95, 14.03) | 16.80 (14.97, 18.63) | 3.81 (1.71, 5.91) | 1.29 (1.12, 1.47) |
|  | 2018 | 14.04 (12.90, 15.18) | 15.83 (14.08, 17.57) | 1.79 (-0.29, 3.87) | 1.13 (0.97, 1.28) |
|  | 2019 | 13.00 (11.85, 14.16) | 15.50 (13.64, 17.36) | 2.50 (0.31, 4.69) | 1.19 (1.01, 1.37) |
| Insufficient physical activity | 2004 | 38.46 (36.79, 40.13) | 40.80 (39.06, 42.54) | 2.34 (-0.06, 4.75) | 1.06 (1.00, 1.13) |
|  | 2005 | 37.91 (36.52, 39.31) | 40.50 (38.76, 42.24) | 2.59 (0.37, 4.81) | 1.07 (1.01, 1.13) |
|  | 2006 | 36.63 (34.99, 38.26) | 38.22 (36.14, 40.29) | 1.59 (-1.04, 4.22) | 1.04 (0.97, 1.12) |
|  | 2007 | 36.69 (34.98, 38.40) | 36.28 (34.14, 38.42) | -0.41 (-3.10, 2.29) | 0.99 (0.92, 1.06) |
|  | 2008 | 35.80 (34.21, 37.40) | 37.61 (35.55, 39.67) | 1.81 (-0.77, 4.38) | 1.05 (0.98, 1.12) |
|  | 2009 | 35.46 (33.98, 36.94) | 34.33 (32.66, 36.01) | -1.12 (-3.35, 1.11) | 0.97 (0.91, 1.03) |
|  | 2010 | 35.56 (33.91, 37.22) | 37.15 (35.28, 39.01) | 1.58 (-0.89, 4.05) | 1.04 (0.97, 1.12) |
|  | 2011 | 36.85 (35.16, 38.55) | 37.12 (35.05, 39.19) | 0.27 (-2.41, 2.94) | 1.01 (0.93, 1.08) |
|  | 2012 | 39.77 (37.16, 42.38) | 40.30 (36.60, 43.99) | 0.53 (-4.01, 5.06) | 1.01 (0.90, 1.13) |
|  | 2013 | 35.88 (34.47, 37.30) | 41.26 (39.01, 43.51) | 5.38 (2.72, 8.03) | 1.15 (1.07, 1.23) |
|  | 2014 | 32.15 (30.65, 33.65) | 37.09 (34.70, 39.48) | 4.94 (2.12, 7.76) | 1.15 (1.06, 1.25) |
|  | 2015 | 32.15 (30.06, 34.24) | 35.12 (32.74, 37.51) | 2.97 (-0.21, 6.15) | 1.09 (0.99, 1.20) |
|  | 2016 | 32.10 (30.67, 33.52) | 37.45 (34.75, 40.16) | 5.36 (2.28, 8.43) | 1.17 (1.07, 1.27) |
|  | 2017 | 32.39 (30.95, 33.83) | 37.26 (34.77, 39.75) | 4.87 (2.02, 7.72) | 1.15 (1.06, 1.24) |
|  | 2018 | 29.97 (28.52, 31.43) | 37.84 (35.28, 40.41) | 7.87 (4.90, 10.84) | 1.26 (1.16, 1.37) |
|  | 2019 | 29.87 (28.25, 31.50) | 33.90 (31.25, 36.54) | 4.02 (0.93, 7.12) | 1.13 (1.03, 1.24) |
| Insufficient fruit and/or vegetable consumption | 2004 | 78.73 (77.35, 80.11) | 79.62 (78.27, 80.97) | 0.89 (-1.03, 2.82) | 1.01 (0.99, 1.04) |
|  | 2005 | 76.64 (75.48, 77.81) | 73.72 (72.30, 75.15) | -2.92 (-4.76, -1.08) | 0.96 (0.94, 0.99) |
|  | 2006 | 73.78 (72.31, 75.25) | 73.22 (71.42, 75.01) | -0.56 (-2.88, 1.75) | 0.99 (0.96, 1.02) |
|  | 2007 | 73.45 (72.01, 74.89) | 71.98 (70.31, 73.65) | -1.47 (-3.73, 0.79) | 0.98 (0.95, 1.01) |
|  | 2008 | 72.56 (71.10, 74.03) | 72.60 (70.92, 74.28) | 0.04 (-2.15, 2.23) | 1.00 (0.97, 1.03) |
|  | 2009 | 71.11 (69.70, 72.51) | 70.33 (68.67, 72.00) | -0.77 (-2.95, 1.40) | 0.99 (0.96, 1.02) |
|  | 2010 | 73.11 (71.61, 74.60) | 71.08 (69.36, 72.80) | -2.03 (-4.30, 0.24) | 0.97 (0.94, 1.00) |
|  | 2011 | 73.92 (72.43, 75.42) | 72.55 (70.63, 74.47) | -1.38 (-3.81, 1.06) | 0.98 (0.95, 1.01) |
|  | 2012 | 73.82 (71.81, 75.83) | 73.52 (70.53, 76.51) | -0.31 (-3.90, 3.29) | 1.00 (0.95, 1.04) |
|  | 2013 | 73.45 (72.10, 74.79) | 75.52 (73.72, 77.32) | 2.07 (-0.18, 4.32) | 1.03 (1.00, 1.06) |
|  | 2014 | 75.44 (74.07, 76.81) | 72.76 (70.67, 74.85) | -2.68 (-5.18, -0.18) | 0.96 (0.93, 1.00) |
|  | 2015 | 78.69 (76.95, 80.43) | 77.70 (75.54, 79.85) | -1.00 (-3.76, 1.77) | 0.99 (0.95, 1.02) |
|  | 2016 | 76.69 (75.31, 78.07) | 77.99 (75.86, 80.12) | 1.29 (-1.24, 3.83) | 1.02 (0.98, 1.05) |
|  | 2017 | 78.79 (77.49, 80.09) | 78.89 (76.82, 80.97) | 0.10 (-2.35, 2.55) | 1.00 (0.97, 1.03) |
|  | 2018 | 81.16 (79.87, 82.45) | 81.24 (79.38, 83.09) | 0.08 (-2.18, 2.34) | 1.00 (0.97, 1.03) |
|  | 2019 | 82.00 (80.60, 83.39) | 80.95 (78.87, 83.04) | -1.04 (-3.55, 1.46) | 0.99 (0.96, 1.02) |
| Daily sugar- sweetened beverage consumption | 2004 | - | - | - | - |
|  | 2005 | - | - | - | - |
|  | 2006 | 30.98 (29.36, 32.59) | 27.02 (25.06, 28.98) | -3.96 (-6.50, -1.42) | 0.87 (0.79, 0.95) |
|  | 2007 | 27.51 (26.06, 28.96) | 27.46 (25.62, 29.31) | -0.05 (-2.41, 2.31) | 1.00 (0.91, 1.08) |
|  | 2008 | 27.07 (25.48, 28.67) | 29.04 (27.19, 30.89) | 1.97 (-0.41, 4.34) | 1.07 (0.98, 1.16) |
|  | 2009 | 29.33 (27.71, 30.94) | 30.73 (28.74, 32.72) | 1.40 (-1.15, 3.95) | 1.05 (0.96, 1.14) |
|  | 2010 | 26.97 (25.10, 28.83) | 27.08 (25.03, 29.13) | 0.11 (-2.60, 2.83) | 1.00 (0.90, 1.11) |
|  | 2011 | - | - | - | - |
|  | 2012 | 22.48 (20.80, 24.17) | 25.61 (23.19, 28.03) | 3.13 (0.17, 6.09) | 1.14 (1.00, 1.28) |
|  | 2013 | - | - | - | - |
|  | 2014 | 22.21 (20.82, 23.61) | 24.92 (22.64, 27.20) | 2.70 (0.02, 5.38) | 1.12 (1.00, 1.25) |
|  | 2015 | 19.15 (17.51, 20.80) | 23.22 (20.80, 25.63) | 4.06 (1.14, 6.99) | 1.21 (1.05, 1.38) |
|  | 2016 | 20.10 (18.79, 21.41) | 23.85 (21.35, 26.36) | 3.75 (0.92, 6.58) | 1.19 (1.04, 1.33) |
|  | 2017 | 20.01 (18.71, 21.32) | 24.98 (22.49, 27.46) | 4.96 (2.16, 7.77) | 1.25 (1.10, 1.40) |
|  | 2018 | 20.19 (18.84, 21.53) | 24.91 (22.61, 27.21) | 4.72 (2.05, 7.39) | 1.23 (1.09, 1.37) |
|  | 2019 | 20.08 (18.57, 21.59) | 24.84 (22.24, 27.45) | 4.76 (1.75, 7.77) | 1.24 (1.08, 1.40) |
| High total lifestyle risk^a^ | 2004 | 49.81 (48.03, 51.59) | 52.22 (50.38, 54.05) | 2.41 (-0.14, 4.96) | 1.05 (1.00, 1.10) |
|  | 2005 | 47.04 (45.56, 48.53) | 49.53 (47.73, 51.32) | 2.48 (0.15, 4.81) | 1.05 (1.00, 1.10) |
|  | 2006 | 44.05 (42.29, 45.80) | 48.27 (46.07, 50.48) | 4.23 (1.42, 7.04) | 1.10 (1.03, 1.16) |
|  | 2007 | 43.54 (41.79, 45.30) | 44.39 (42.16, 46.63) | 0.85 (-2.00, 3.69) | 1.02 (0.95, 1.09) |
|  | 2008 | 43.95 (42.21, 45.68) | 45.18 (43.00, 47.37) | 1.23 (-1.58, 4.05) | 1.03 (0.96, 1.09) |
|  | 2009 | 43.37 (41.77, 44.97) | 43.73 (41.80, 45.66) | 0.36 (-2.14, 2.86) | 1.01 (0.95, 1.07) |
|  | 2010 | 42.20 (40.42, 43.99) | 44.75 (42.73, 46.78) | 2.55 (-0.14, 5.24) | 1.06 (0.99, 1.13) |
|  | 2011 | 42.91 (41.12, 44.70) | 45.10 (42.84, 47.35) | 2.18 (-0.70, 5.06) | 1.05 (0.98, 1.12) |
|  | 2012 | 45.11 (42.52, 47.70) | 46.96 (43.33, 50.58) | 1.85 (-2.57, 6.27) | 1.04 (0.94, 1.14) |
|  | 2013 | 42.05 (40.53, 43.56) | 48.94 (46.57, 51.31) | 6.89 (4.08, 9.71) | 1.16 (1.09, 1.23) |
|  | 2014 | 41.58 (39.94, 43.21) | 44.01 (41.46, 46.56) | 2.43 (-0.59, 5.45) | 1.06 (0.98, 1.13) |
|  | 2015 | 40.65 (38.33, 42.98) | 45.37 (42.58, 48.16) | 4.71 (1.08, 8.35) | 1.12 (1.02, 1.21) |
|  | 2016 | 40.83 (39.26, 42.40) | 47.79 (45.01, 50.57) | 6.96 (3.77, 10.16) | 1.17 (1.09, 1.25) |
|  | 2017 | 41.47 (39.90, 43.04) | 50.41 (47.75, 53.08) | 8.95 (5.85, 12.05) | 1.22 (1.14, 1.29) |
|  | 2018 | 42.22 (40.60, 43.84) | 49.16 (46.48, 51.84) | 6.94 (3.80, 10.08) | 1.16 (1.09, 1.24) |
|  | 2019 | 42.29 (40.46, 44.13) | 48.11 (45.24, 50.99) | 5.82 (2.41, 9.23) | 1.14 (1.05, 1.22) |
| High total lifestyle risk (supplementary analyses)^b^ | 2004 | - | - | - | - |
|  | 2005 | - | - | - | - |
|  | 2006 | 56.49 (54.75, 58.23) | 58.62 (56.54, 60.70) | 2.13 (-0.58, 4.84) | 1.04 (0.99, 1.09) |
|  | 2007 | 55.30 (53.60, 56.99) | 55.41 (53.40, 57.43) | 0.12 (-2.46, 2.69) | 1.00 (0.96, 1.05) |
|  | 2008 | 54.22 (52.46, 55.99) | 56.19 (54.11, 58.28) | 1.97 (-0.75, 4.69) | 1.04 (0.99, 1.09) |
|  | 2009 | 55.31 (53.66, 56.96) | 56.06 (54.12, 58.00) | 0.75 (-1.79, 3.30) | 1.01 (0.97, 1.06) |
|  | 2010 | 53.28 (51.39, 55.17) | 55.57 (53.48, 57.66) | 2.29 (-0.53, 5.11) | 1.04 (0.99, 1.10) |
|  | 2011 | - | - | - | - |
|  | 2012 | 53.77 (51.22, 56.32) | 56.54 (52.92, 60.17) | 2.77 (-1.68, 7.22) | 1.05 (0.97, 1.14) |
|  | 2013 | - | - | - | - |
|  | 2014 | 50.43 (48.79, 52.07) | 53.20 (50.69, 55.71) | 2.77 (-0.22, 5.77) | 1.06 (0.99, 1.12) |
|  | 2015 | 47.89 (45.49, 50.29) | 54.18 (51.40, 56.96) | 6.29 (2.61, 9.97) | 1.13 (1.05, 1.21) |
|  | 2016 | 49.75 (48.15, 51.35) | 55.70 (52.98, 58.42) | 5.95 (2.79, 9.11) | 1.12 (1.05, 1.19) |
|  | 2017 | 49.98 (48.39, 51.58) | 58.62 (56.10, 61.13) | 8.63 (5.65, 11.62) | 1.17 (1.11, 1.24) |
|  | 2018 | 50.37 (48.72, 52.01) | 58.32 (55.73, 60.90) | 7.95 (4.88, 11.01) | 1.16 (1.09, 1.22) |
|  | 2019 | 50.64 (48.78, 52.49) | 56.96 (54.15, 59.77) | 6.32 (2.97, 9.68) | 1.12 (1.06, 1.19) |

Abbreviations: ARIA+, Accessibility-Remoteness Index of Australia Plus; CI, confidence interval.

* Risk differences and risk ratios are compared with the base category of 'metro'.

^a^ Defined as engaging in two or more lifestyle risk behaviours, based on the following four individual risk behaviours: excessive alcohol consumption, insufficient physical activity, insufficient fruit and/or vegetable consumption, and current smoking.

^b^ Defined as engaging in two or more lifestyle risk behaviours, based on the following five individual risk behaviours: excessive alcohol consumption, insufficient physical activity, insufficient fruit and/or vegetable consumption, current smoking, and daily sugar-sweetened beverage consumption.
