# Supplementary material for: Sixteen-year trends in multiple lifestyle risk behaviours by socioeconomic status from 2004 to 2019 in New South Wales, Australia
Source: PLOS Glob Public Health. 2023 Feb 15;3(2):e0001606. doi: 10.1371/journal.pgph.0001606 (PMC10021655; doi:10.1371/journal.pgph.0001606)
Supplement: Attachment — Submitted filename: Response to reviewers_Nguyen.doc [file pgph.0001606.s010.doc]

|  | |  |     **Prevention Research Collaboration**  **Sydney School of Public Health** | | |
| --- | --- | --- | --- | --- | --- |
|  | ABN 15 211 513 464 | |  |  |  |
|  |  | | Level 6, The Charles Perkins Centre (D17)  Sydney NSW 2006 AUSTRALIA | | |
|  |  |  | Phone:  Fax: | +61 2 8627 1865  +61 2 8627 1602 |  |
|  |  |  | Email: | thanh-binh.nguyen-duy@sydney.edu.au | |

19^th^ January, 2023

Dear Editor,

We thank you for the opportunity to revise and resubmit our paper “Sixteen-year trends in multiple lifestyle risk behaviours by socioeconomic status from 2004 to 2019 in New South Wales, Australia”. We are grateful to the reviewers for their constructive feedback. Please find below our response to the comments from the reviewers and journal. Our response is in Italics. Any changes made to the paper can be seen as tracked changes in the “Revised manuscript with tracked changes” document. Pages and line numbers mentioned in our response relate to the “Revised manuscript with tracked changes”.

**Reviewer comments**

**Reviewer 1**

I enjoyed reading this well-written, important paper. The methodology was well prepared, the results well described and argued in the discussion. The conclusions are relevant to the field of public health. The entire paper is supported with the relevant literature. I wish authors all the best and look forward to seeing the paper published.

*Thank you for this very encouraging feedback.*

**Reviewer 2**

1. The authors sophisticatedly and carefully examine long-term changes in the prevalence of various behavioral risks and the socioeconomic disparities therein. The methods used are appropriate, and the authors’ findings are inspiring. This is a very well-written manuscript. However, I have some questions about modeling decisions and interpretations that, if clarified, would strengthen the paper. These, along with more minor questions/suggestions, are listed below.

*We appreciate your overall positive feedback and specific comments to improve our manuscript.*

2. Could the authors provide more justifications for why ARIA+ can be a measure of socioeconomic status? For example, although there are fewer healthcare services in rural areas, whether one is disadvantaged depends on his/her wealth. That is, wealthy people have cars and may buy houses in rural areas, whereas poor people may live in the cities, but poverty may restrict their access to health-related resources.

*Thank you for your question. The Accessibility-Remoteness Index of Australia Plus (ARIA+) is a commonly used index to measure geographical remoteness in Australia. The index provides accessibility/remoteness values for all of Australia based on road distance measurements from over 12,000 populated localities to the nearest Service Centres in five size categories based on population size (*[*https://able.adelaide.edu.au/hugo-centre/services/aria*](https://able.adelaide.edu.au/hugo-centre/services/aria)*). It is meant to be an area-level geographical index rather than an individual-level indicator. In Australia, remoteness is strongly linked with socioeconomic disadvantages (Australian Institute of Health and Welfare, Rural and Remote Health, July 2022;* [*https://www.aihw.gov.au/reports/rural-remote-australians/rural-and-remote-health*](https://www.aihw.gov.au/reports/rural-remote-australians/rural-and-remote-health)*), particularly in terms of access to services, and is commonly considered as an important socioeconomic variable. Numerous studies have linked remoteness with a broad range of health outcomes, such as cancer survival (Jong et al. 2004, Remoteness of residence and survival from cancer in New South Wales), obesity (Keramat et al. 2021, Trends in the prevalence of adult overweight and obesity in Australia, and its association with geographic remoteness) and premature mortality (Australian Institute of Health and Welfare, Rural and Remote Health, July 2022;* [*https://www.aihw.gov.au/reports/rural-remote-australians/rural-and-remote-health*](https://www.aihw.gov.au/reports/rural-remote-australians/rural-and-remote-health)*). Remoteness was considered as part of a broader range of socioeconomic status indicators. In the manuscript, we have now specified that geographical remoteness, “has been linked with socioeconomic disadvantages” (page 6, line 119) and provided a reference.*

3. Could the authors elaborate why area-level indicators are better than individual-level indicators for capturing respondents’ socioeconomic status? Could the authors explain why SEIFA is a better measure than individual-level wealth/income?

*In our study, we used both an individual-level indicator (educational attainment which is widely used in the literature) and area-level indicators (socioeconomic disadvantage and geographic remoteness) to capture respondents’ socioeconomic status, as described on page 6 (lines 106-108). We could not use individual-level income due to the large amount of missing data and misclassification bias for this age group (e.g., for many retired participants, the main contributor to their wealth was asset, which we could not capture). SEIFA is one of the most commonly used indicators of socioeconomic status in Australia and it was designed to capture various area-level attributes, such as unemployment, income, occupations, all of which are relevant to health behaviours. As a composite scale, SEIFA captures additional information that could not be measured by individual-level variables alone. All of these indicators (education, SEIFA, ARIA+) are important to consider as they measure different aspects of socioeconomic status (as stated on page 6, line 108). Individual-level socioeconomic status alone provides a partial picture. SEIFA and ARIA+ were included to more completely cover the potential impacts of disadvantage, which can be at either an individual or societal level, or both. In the manuscript, we have now specified that SEIFA IRSD is a “commonly used indicator of socioeconomic status in Australia” (page 6, line 113).*

4. P.7-8: Could the authors elaborate why controlling for both immigration status and linguistic backgrounds is necessary? I am wondering why “whether they spoke a language other than English at home” matters, since it seems not necessarily related to respondents’ English proficiency.

*The effects of country of birth and speaking a language other than English at home are not necessarily the same and they are often not highly correlated in population settings. For example, a large proportion of immigrants in Australia are from the UK or New Zealand. They would be classified as immigrants based on the country of birth question. Contrarily, some second-generation foreign-born Australians may still speak their native language at home and maintain a strong cultural tie. Neither country of birth or language spoken at home is a perfect indicator, but both together is better than either alone. In fact, both are routinely used by governments in Australia (e.g., Australian Bureau of Statistics) as indicators for cultural and linguistic diversity. We have therefore included both as covariates. In the manuscript, we have now added that: “Country of birth and whether a language other than English is spoken at home are routinely used by governments in Australia as indicators for cultural and linguistic diversity” (page 8, lines 157-159).*

5. P.8: Is the distribution of the lifestyle risk index right-skewed? Would using poisson or negative binominal regression change the results?

*We did not treat the lifestyle risk index as continuous. The lifestyle risk index was dichotomised based on reporting at least two of the risk behaviours. While a count model is possible, it would have a very different meaning and interpretation to the current analysis. In the manuscript, we have now added “dichotomous” to the following sentence (page 7, line 143): “Finally, a dichotomous lifestyle risk index was developed…”.*

6. P.15: The authors said, “For current smoking, the prevalence decreased for all education subgroups

over time”. However, in Figure 2, CIs for the smoking prevalence in the least educated group largely overlap across years. This is also the case for insufficient physical activity and lifestyle risk index.

*We have now tempered the language in the manuscript and modified the sentence on page 15 (lines 269-271) to: "For current smoking, the prevalence decreased for the two least disadvantaged education subgroups over time. There was a suggestion of a decrease for the most disadvantaged subgroup, although it was not significant."*

*For insufficient physical activity, we have added “suggested” (underlined) in the following sentence (page 16, line 296): “For physical activity, despite the suggested decline in insufficient physical activity overall, …”.*

*For the lifestyle risk index, we had mentioned in the manuscript that “… the prevalence of an overall high-risk lifestyle (having two or more of the above risk factors) remained relatively stable in the most disadvantaged subgroup over time…” (page 17, lines 327-329).*

7. P16: It seems that the authors did not interpret results regarding the remoteness differences in excessive alcohol consumption.

*Thank you for pointing this out. We have now interpreted the results in relation to remoteness differences in excessive alcohol consumption. We have added that “Between 2004 and 2019, there was no clear increasing or decreasing trend over time in absolute and relative inequalities in excessive alcohol consumption by education. There was a relatively stable inequality by social disadvantage, and no substantial inequality by remoteness.” (page 16, lines 292-295).*

8. P20: It seems to me that the prevalence of insufficient fruit/vegetable consumption tends to increase over time, which is more evident in Figure 3-5. Could the authors add more discussions on what potential reasons would be even if there is a state-wide healthy eating program?

*In relation to the observed increase in prevalence of insufficient fruit/vegetable consumption over time, we had written in the manuscript that “For insufficient fruit and vegetable consumption, the prevalence increased in all subgroups based on education, area-level disadvantage and remoteness from 2009 onwards” (page 17, lines 309-310).*

*Multiple factors could have contributed to the low prevalence of sufficient fruit and vegetable consumption. For example, campaigns promoting fruit and vegetable consumption may need to run for multiple years to achieve population impact (Grunseit et al. 2016, Mass media campaigns addressing physical activity, nutrition and obesity in Australia 1996 – 2015: an updated narrative review, The Australian Prevention Partnership Centre). Higher costs of fruit and vegetables relative to household income could be a contributing factor to lower consumption levels, particularly among people from lower socioeconomic status (Chapman et al. 2017, Exploring perceptions and beliefs about the cost of fruit and vegetables and whether they are barriers to higher consumption; NSW Council of Social Service [NCOSS], Access to Healthy Food - NCOSS Cost of Living Report, September 2018). Preferences for other foods and poor recognition of inadequate fruit/vegetable consumptions may also be contributing factors (Chapman et al. 2016, Time to address continued poor vegetable intake in Australia for prevention of chronic disease).*

*In the Discussion, the following sentence h*as *been added along with relevant references (page 20, lines 378-380): “This may be due to many factors including the cost of fruit and vegetables relative to household income, food preferences and poor recognition of inadequate fruit or vegetable consumption.”*

9. P21: Regarding the “counterintuitive” results of excessive alcohol assumption, it is documented that the relationship between drinking behavior and health outcomes depends on the quality and types of alcohol. Perhaps the authors can interpret observed patterns in this way.

*Thank you for this suggestion. Unfortunately, the NSW PHS survey data did not ask participants about the types of alcoholic beverages consumed.*

10. Figure 1: Could the authors add a line for the means of prevalence so that readers can easily identify whether the prevalence increases or decreases?

*We have now added a line that represents the mean prevalence over the study period in Revised Figure 1 and added a caption describing what it represents (page 13, line 230): “The dotted horizontal line represents the mean prevalence over the years 2014-2019”.*

**Journal requirements**

We do not publish any copyright or trademark symbols that usually accompany proprietary names, eg (R), (C), or TM (e.g. next to drug or reagent names). Please remove all instances of trademark/copyright symbols throughout the text, including R on page 20.

Please review your reference list to ensure that it is complete and correct. If you have cited papers that have been retracted, please include the rationale for doing so in the manuscript text, or remove these references and replace them with relevant current references. Any changes to the reference list should be mentioned in the rebuttal letter that accompanies your revised manuscript. If you need to cite a retracted article, indicate the article’s retracted status in the References list and also include a citation and full reference for the retraction notice.

*Thank you for reminding us of the journal requirement to remove all instances of trademark/copyright symbols throughout the text. We have checked the text and removed the R symbols on pages 20-21 (lines 371 and 391). We have also reviewed the reference list to make sure that it is complete and correct. Four references (numbers 24, 51, 52, 53) have been added to the reference list in relation to revisions made to the manuscript in response to reviewers’ comments.*

Yours sincerely

Dr Binh Nguyen,

Research Fellow

Prevention Research Collaboration, Sydney School of Public Health

University of Sydney
